# Single-cell RNA sequencing identifies an *Il1rn*^+^*/Trem1*^+^ macrophage subpopulation as a cellular target for mitigating the progression of thoracic aortic aneurysm and dissection

**DOI:** 10.1038/s41421-021-00362-2

**Published:** 2022-02-08

**Authors:** Xuanyu Liu, Wen Chen, Guoyan Zhu, Hang Yang, Wenke Li, Mingyao Luo, Chang Shu, Zhou Zhou

**Affiliations:** 1 State Key Laboratory of Cardiovascular Disease, Fuwai Hospital, National Center for Cardiovascular Diseases, Chinese Academy of Medical Sciences and Peking Union Medical College, Beijing, China; 2 Beijing Key Laboratory for Molecular Diagnostics of Cardiovascular Diseases, Center of Laboratory Medicine, Fuwai Hospital, Beijing, China; 3 Center of Vascular Surgery, Fuwai Hospital, Beijing, China; 4 Department of Vascular Surgery, Fuwai Yunnan Cardiovascular Hospital, Affiliated Cardiovascular Hospital of Kunming Medical University, Kunming, Yunnan China

**Keywords:** Innate immunity, Mechanisms of disease

## Abstract

Thoracic aortic aneurysm and dissection (TAAD) is a life-threatening condition characterized by medial layer degeneration of the thoracic aorta. A thorough understanding of the regulator changes during pathogenesis is essential for medical therapy development. To delineate the cellular and molecular changes during the development of TAAD, we performed single-cell RNA sequencing of thoracic aortic cells from β-aminopropionitrile-induced TAAD mouse models at three time points that spanned from the early to the advanced stages of the disease. Comparative analyses were performed to delineate the temporal dynamics of changes in cellular composition, lineage-specific regulation, and cell–cell communications. Excessive activation of stress-responsive and Toll-like receptor signaling pathways contributed to the smooth muscle cell senescence at the early stage. Three subpopulations of aortic macrophages were identified, i.e., *Lyve1*^+^ resident-like, *Cd74*^high^ antigen-presenting, and *Il1rn*^+^/*Trem1*^+^ pro-inflammatory macrophages. In both mice and humans, the pro-inflammatory macrophage subpopulation was found to represent the predominant source of most detrimental molecules. Suppression of macrophage accumulation in the aorta with Ki20227 could significantly decrease the incidence of TAAD and aortic rupture in mice. Targeting the *Il1rn*^+^/*Trem1*^+^ macrophage subpopulation via blockade of Trem1 using mLR12 could significantly decrease the aortic rupture rate in mice. We present the first comprehensive analysis of the cellular and molecular changes during the development of TAAD at single-cell resolution. Our results highlight the importance of anti-inflammation therapy in TAAD, and pinpoint the macrophage subpopulation as the predominant source of detrimental molecules for TAAD. Targeting the *IL1RN*^+^/*TREM1*^+^ macrophage subpopulation via blockade of TREM1 may represent a promising medical treatment.

## Introduction

Thoracic aortic aneurysm and dissection (TAAD) is a life-threatening condition occurring in ~6–10 cases every 100,000 people^1^. Medial layer degeneration of the thoracic aorta is the defining histopathologic feature of TAAD, which is characterized by vascular smooth muscle cell (SMC) loss, elastic fiber fragmentation, and extracellular matrix (ECM) degradation in the aortic wall, which might eventually lead to rupture and death^2^. TAAD can be syndromic (e.g., Marfan syndrome; MFS), familial nonsyndromic, or sporadic^3^. In contrast with the extensive studies on abdominal aortic aneurysm and dissection (AAAD), our understanding of the pathogenesis mechanisms of TAAD remains limited, especially for sporadic TAAD, which constitutes the majority (80%) of cases^4^. Despite recent advances in surgery, effective targeted medical therapies to prevent or mitigate the progression of this disease are still lacking.

Our current knowledge on the molecular mechanisms of TAAD primarily stems from genetic studies and genetic mouse models^4^. Several mutated genes that predispose an individual to TAAD have been discovered, which mainly encode proteins in the elastin-contractile unit (the structural unit linking the elastin lamellae to the SMCs) and TGF beta signaling^5^. The first gene identified for heritable TAAD is *FBN1* whose mutation causes MFS^6^. *Fbn1* mutation mouse model has been broadly used to study the molecular mechanisms of MFS-associated TAAD^7–9^. Nevertheless, the knowledge obtained through genetic studies and genetic models might be limited in terms of the generalizability to a greater population of cases with sporadic TAAD. Unlike heritable TAAD, sporadic TAAD is more influenced by environmental factors rather than genetic predisposition, e.g., mechanical stress triggered by hypertension, a crucial risk factor for sporadic TAAD^5^. Notably, administration of β-aminopropionitrile (BAPN; a lysyl oxidase inhibitor) to mice disrupts the crosslinking of collagen and elastin, which subjects the aortic wall to elevated mechanical stress and ultimately leads to TAAD^10^. Therefore, the BAPN-induced TAAD mouse model is suitable for studying the pathogenesis of mechanical stress-induced sporadic TAAD^11^.

Understanding the sequence of the molecular events during the initiation and progression of TAAD is of fundamental importance for identifying molecular and cellular targets for medical therapy development. However, this cannot be attained solely based on human patient samples, since they normally represent an advanced stage of the disease. Time-series single-cell RNA sequencing (scRNA-seq) of cells from mouse models has recently emerged as a powerful tool to analyze the alterations in cellular composition, lineage-specific regulation, and cell–cell communications during the development of heart diseases such as heart failure^12^ and atherosclerosis^13^. A recent study reported the transcriptomic alterations in aortic cells from human patients with advanced-stage ascending thoracic aortic aneurysm using scRNA-seq^14^. However, transcriptomic changes during the initiation and progression of TAAD remain largely understudied.

Here, we performed scRNA-seq of thoracic aortic cells obtained from BAPN-induce TAAD mouse models at three time points that spanned from the early to the advanced stages of the disease. We aimed to delineate the temporal dynamics of changes in cellular composition, lineage-specific regulation, and cell–cell communications during the development of TAAD.

## Results

### Time-series scRNA-seq reveals cellular heterogeneity in the thoracic aorta during the development of TAAD

**Fig. 1:**
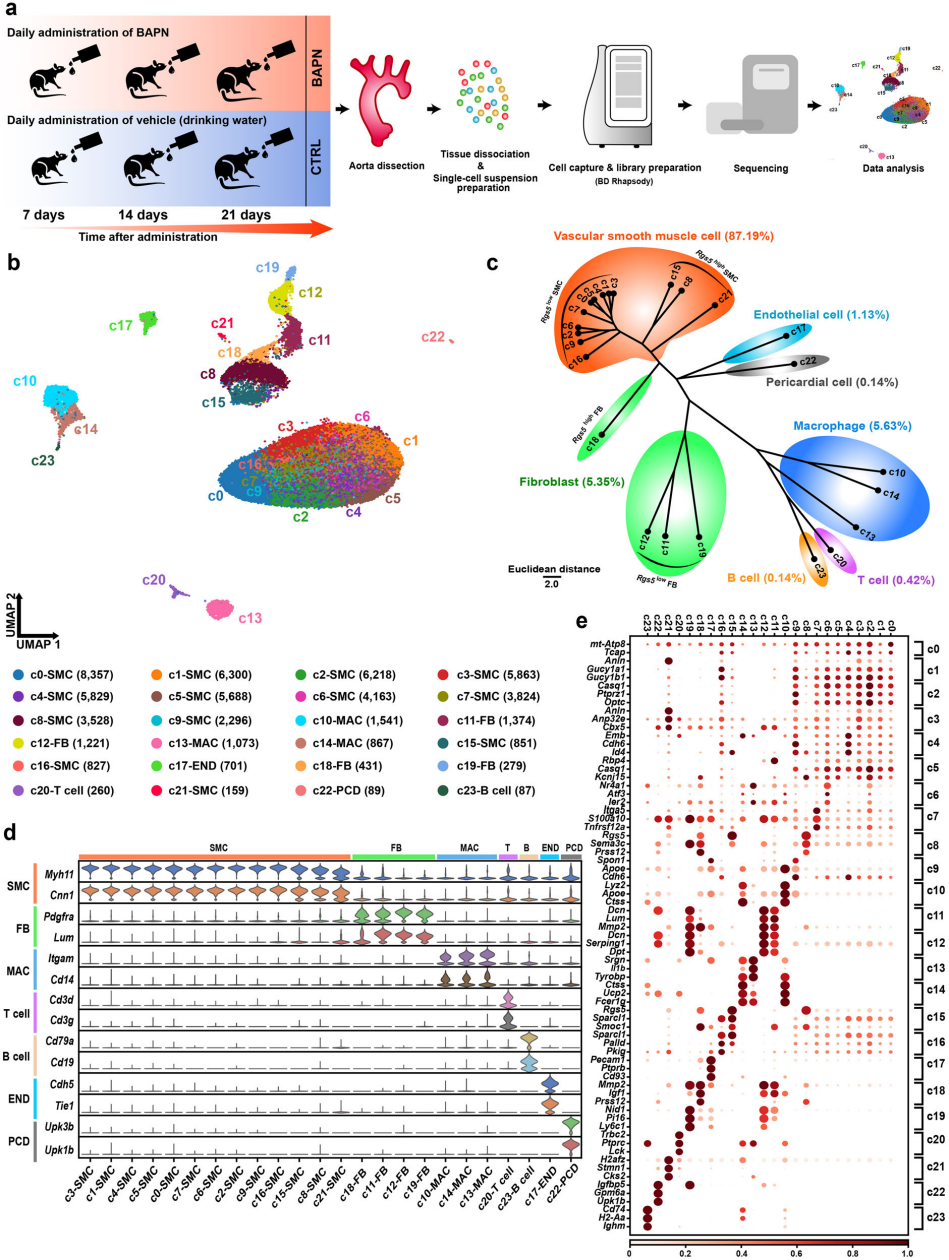
scRNA-seq reveals cellular heterogeneity in the thoracic aorta during the development of TAAD. **a** Schematic representation of the scRNA-seq procedure. The ascending aorta and aortic arch were collected at three time points during the induction, i.e., after 7, 14, and 21 days of BAPN or vehicle (drinking water) administration. Tissues from six mice were pooled for each time point for scRNA-seq. CTRL, control. **b** Unbiased clustering of 61,826 cells from all six samples reveals 24 cellular clusters. The number in the parenthesis indicates the cell count. **c** Hierarchical clustering of the clusters based on the average expression of the 2000 most variable genes. **d** Expression of the established marker genes specific for each lineage in each cluster. **e** Representative molecular signatures for each cell cluster. The area of the circles indicates the proportion of cells expressing the gene, and the color intensity reflects the expression intensity. EDO, endothelial cell; FB, fibroblast; MAC, macrophage; PCD, pericardial cell; SMC, smooth muscle cell.

We used a mouse model induced by administering BAPN to study the initiation and progression of TAAD. To delineate cellular heterogeneity in the thoracic aorta in vivo during the development of TAAD, the ascending aorta and aortic arch were harvested at three time points during the induction, i.e., after 7, 14, and 21 days of BAPN administration (Fig. 1a). To exclude the potential effects of growth and development since the mice were young (3-week-old), the tissues of mice administrated vehicle (drinking water) were also collected at the corresponding time points as a control. Elastin staining showed a continuous alteration in histological properties of the thoracic aorta (Supplementary Fig. S1). Compared with the control, the aorta displayed no visible changes after 7 days of BAPN administration, which thus represented the early stage of pathogenesis. After 14 days of BAPN administration, the aortic wall showed disarrayed and straightening elastic fibers, indicating an initiation stage. In contrast with the other two stages, the aortic wall exhibited typical TAAD phenotypes, including fragmented elastic fibers, SMC loss, decreased media thickness, aortic wall dilation, and dissection after 21 days of BAPN administration, thus representing an advanced stage of TAAD.

The aortic tissue was dissociated and then subjected to scRNA-seq using the BD Rhapsody™ platform (Supplementary Table S1). Unbiased clustering of 61,826 cells from all six samples revealed 24 clusters (Fig. 1b). Based on hierarchical clustering (Fig. 1c) and established lineage-specific marker genes (Fig. 1d), these clusters were assigned to seven cell lineages. Representative molecular signatures of these clusters are shown in Fig. 1e and Supplementary Table S2. Typical aortic cell types were discovered, including vascular SMCs (marked by *Myh11* and *Cnn1*)^15^, fibroblasts (marked by *Pdgfra* and *Lum*)^16^, and endothelial cells (*Cdh5* and *Tie1*)^17^. In addition to a few T cells (marked by *Cd3d* and *Cd3g*)^18^ and B cells (marked by *Cd79a* and *Cd19*)^19^, macrophages (marked by *Itgam* and *Cd14*)^20^ were the most abundant immune cells (5.63%). A web-based interface (http://scrnaseqtaad.fwgenetics.org) was developed for further exploration of the dataset.

It is known that vascular SMCs may undergo phenotypic modulation in response to stimuli, which is characterized by downregulation of contractile markers, and upregulation of ECM as well as matrix metalloproteinase (MMP) genes (a synthetic phenotype similar to fibroblasts)^21^. Notably, based on the expression levels of *Rgs5*, the SMC and fibroblast lineages could be subdivided into *Rgs5*^low^ SMCs, *Rgs5*^high^ SMCs, *Rgs5*^low^ fibroblasts, and *Rgs5*^high^ fibroblasts (Fig. 1c and Supplementary Fig. S2a). Strikingly, *Rgs5*^high^ SMCs (c8, c15, and c21) were separated from *Rgs5*^low^ SMCs in the uniform manifold approximation and projection (UMAP) embeddings, and aligned closely to fibroblasts, reflecting distinct cellular states. Differentially expression analysis revealed that, compared to *Rgs5*^low^ SMCs, *Rgs5*^high^ SMCs expressed significantly higher levels of molecular signatures for phenotypically modulated SMCs as reported by recent single-cell studies^7,22^, including *Fn1*, *Lmac3*, *Mmp2*, and *Col1a1* (Supplementary Fig. S2b). Additionally, *Rgs5*^high^ SMCs exhibited downregulated expression of contractile markers including *Acta2*, *Mylk4*, and *Myh11*. Gene Ontology (GO) analysis revealed that the upregulated genes of *Rgs5*^high^ SMCs were enriched for terms related to ECM remodeling and migratory features of modulated SMCs, such as “extracellular matrix organization” and “positive regulation of smooth muscle cell migration” (Supplementary Fig. S2c). The ECM secreting and migratory phenotypes shared by modulated SMCs and fibroblasts may explain their closeness in the UMAP space. Moreover, single-molecule fluorescent in situ hybridization (smFISH) results confirmed the presence of *Rgs5*^high^ SMCs, most of which were located at the outer layers of the tunica media (Supplementary Fig. S2d). Therefore, *Rgs5*^high^ SMCs may represent phenotypically modulated SMCs.

### Differential proportional analysis reveals significantly expanded or contracted cell lineages and clusters associated with the progression of TAAD

**Fig. 2:**
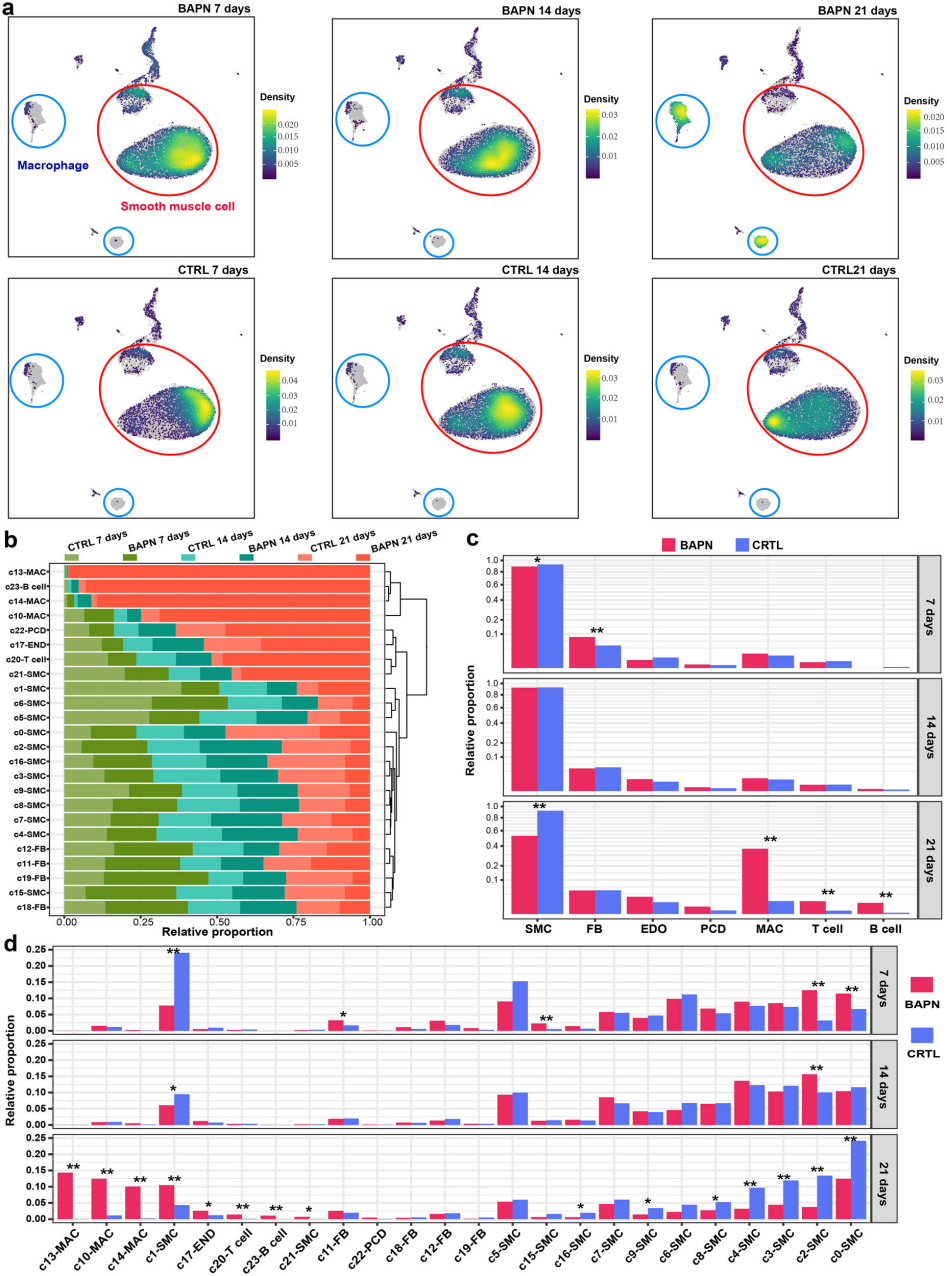
Differential proportional analysis reveals significantly expanded or contracted cell lineages associated with the progression of TAAD. **a** Visualization of cellular density reveals dramatic changes in proportion for smooth muscle cells and macrophages in BAPN vs CTRL. **b** The proportion of cells from each sample in each cluster. **c** The relative proportion of each lineage at each stage. Square root transformation was performed on the *y*-axis. **d** The relative proportion of each cluster at each time point. In **a** and **b**, cells are randomly sampled for equal numbers in each sample (*n* = 7316). In **c** and **d**, a DPA test was performed. *P < 0.05, **P < 0.01; CTRL, control; EDO, endothelial cell; FB, fibroblast; MAC, macrophage; PCD, pericardial cell; SMC, smooth muscle cell.

Cell lineages that significantly changed in relative proportion were probably associated with the pathogenesis of the disease. Visualization of cellular density across the samples demonstrated dramatic changes in relative proportion for some lineages during the induction (Fig. 2a). In line with SMC apoptosis and loss in TAAD^4^, a great contraction of the SMC lineage was observed after 21 days of induction (the advanced stage). Meanwhile, a dramatic expansion of the macrophages occurred after 21 days of induction, indicating enhanced macrophage infiltration at the advanced stage. To quantify the expansion and contraction, the relative proportion of each sample in each cluster was examined (Fig. 2b). Strikingly, macrophage clusters c13 and c14 were expanded with time, reflecting their association with the progression of TAAD. To statistically determine whether the proportional changes were expected by chance, a permutation-based statistical test (differential proportion analysis; DPA) was performed as described previously^23^. At the early stage (after 7 days of BAPN administration), the SMCs had already been significantly contracted (Fig. 2c; DPA test *P* value < 0.05). Notably, the aorta at the advanced stage (after 21 days of BAPN administration) exhibited dramatic changes in the relative proportion of cell lineages, which were characterized by significant contraction of SMCs and expansion of immune cells including macrophages, T, and B cells (Fig. 2c; DPA test *P* value < 0.05). Furthermore, DPA testing at the cluster level revealed that all the three macrophage clusters, especially c13, were significantly expanded at the advanced stage (Fig. 2d). Notably, *Rgs5*^high^ SMC cluster c15 was significantly expanded at the early stage (7 days), which was consistent with its role as phenotypically modulated SMCs in response to stimuli (Fig. 2d). Considering the significant changes in relative proportion, SMC and macrophage, the two largest aortic lineages of non-immune and immune cells, respectively, were underscored in the following analysis.

### Dysregulated pathways in SMCs during the development of TAAD

**Fig. 3:**
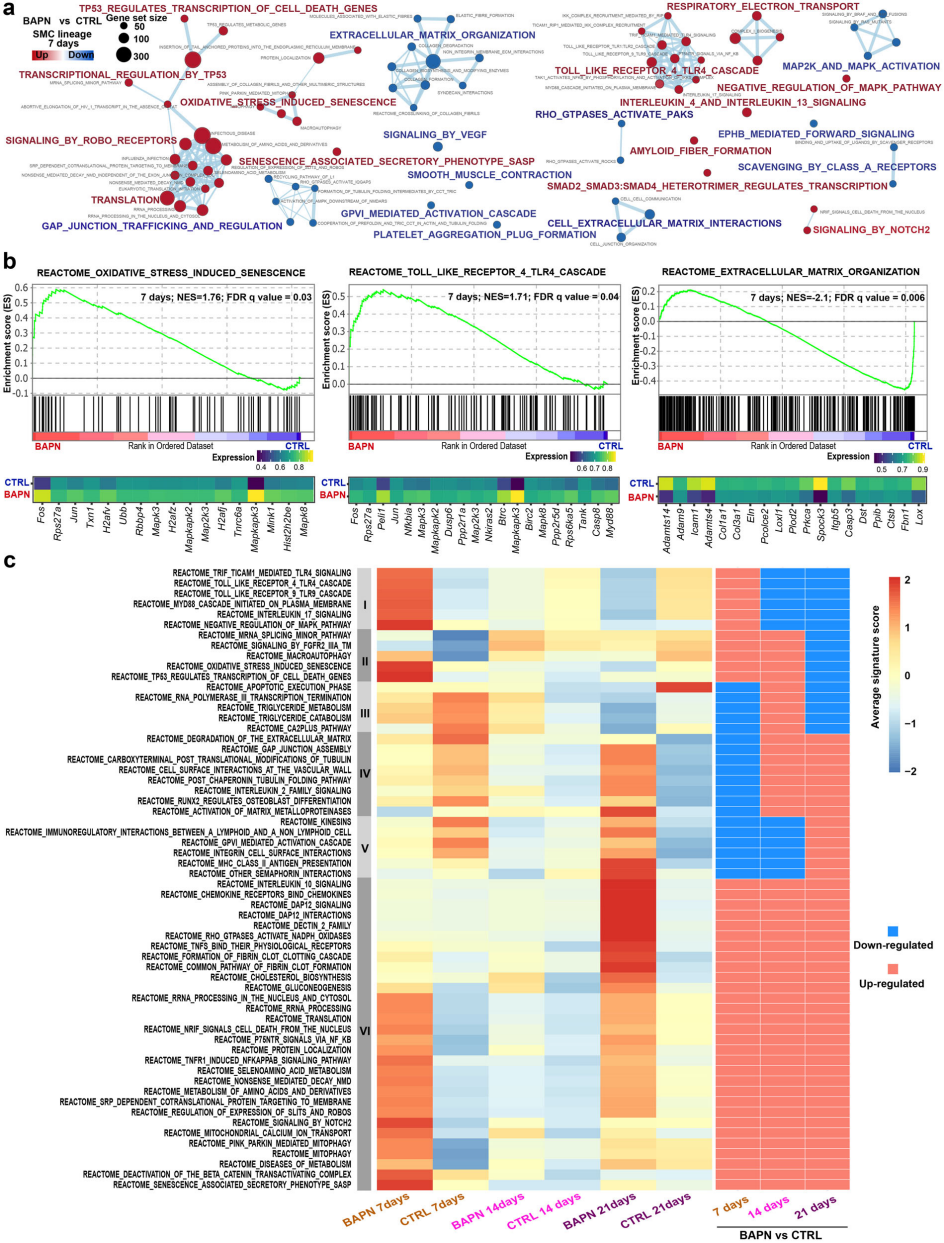
Dysregulated pathways in SMCs during the development of TAAD. **a** Network view of the differentially regulated REACTOME pathways in SMCs at the early stage of TAAD induction. The size of the dot reflects the size of the gene set. The dots in red denote upregulated pathways, and the dots in blue represent downregulated pathways. The significance threshold of the GSEA test was set to an FDR *q* value of 0.05. **b** Enrichment plots (upper panel) and leading-edge gene expression heatmaps (lower panel) for representative pathways dysregulated in SMCs at the early stage of TAAD induction. NES, normalized enrichment score. The vertical lines in the enrichment plot show where the members of the gene set appear in the ranked list of genes. The average expression across cells in each group is shown in the heatmap. **c** The expression dynamics of representative pathways dysregulated during the induction of TAAD. Gene set-based signature scoring was performed using the method implemented in Single-Cell Signature Explorer. The expression activity of a gene set in each sample is represented by the average of the signature scores across cells in the sample.

SMCs represent the major structural component of the aortic wall^3^. We next sought to identify SMC-specific dysregulated pathways during the development of TAAD (Fig. 3 and Supplementary Table S3) via gene set enrichment analysis (GSEA), which facilitates biological interpretation by robustly detecting concordant differences at the gene set level^24^. We first focused on the regulatory alterations in SMCs at the early stage of TAAD (after 7 days of BAPN administration). Consistent with SMC loss in TAAD, cell senescence and death-related pathways were significantly upregulated in SMCs, such as “oxidative stress-induced senescence” and “TP53 regulates transcription of cell death genes” (Fig. 3a; GSEA false discovery rate (FDR) <0.05). Oxidative stress-related genes, e.g., *Txn1*, were upregulated in the BAPN group (Fig. 3b). Endoplasmic reticulum (ER) stress has been suggested to promote SMC apoptosis in TAAD^11,25^. Indeed, an upregulation of the pathway “intrinsic apoptotic signaling pathway in response to ER stress” was observed (Supplementary Fig. S3). Representative ER stress genes, such as *Atf4* and *Ddit3*, were upregulated in BAPN. Toll-like receptor (TLR) 4 pathway, an innate immune signaling pathway, has recently been implicated in aortic homeostasis and pathogenesis^26^. Notably, Toll-like receptor signaling pathways were activated particularly at the early stage, and the representative gene *Myd88* was upregulated (Fig. 3b, c). Collectively, our results highlight the contribution of oxidative stress, ER stress, and sustained activation of Toll-like receptor signaling pathways to the SMC senescence and apoptosis at the initiation stage of TAAD. In addition, as evidenced by the downregulation of the pathway “smooth muscle contraction” (Supplementary Fig. S3), the contractile function of SMCs was weakened. In line with ECM degradation in TAAD, ECM-related pathways, such as “extracellular matrix organization”, were also significantly downregulated (Fig. 3b).

We also examined the regulatory changes at the other two time points (Fig. 3c and Supplementary Fig. S4). The SMCs after 14 days of BAPN administration were characterized by the upregulation of pathways, including “triglyceride metabolism” and “cholesterol biosynthesis”. Notably, the SMCs at the advanced stage (21 days) exhibited pronounced upregulation of immune-related pathways, such as “MHC class II antigen presentation”, “interleukin 10 signaling” and “chemokine receptors bind chemokines”.

### Comparative analysis of gene regulatory networks reveals key molecules of SMCs at the early stage of TAAD

**Fig. 4:**
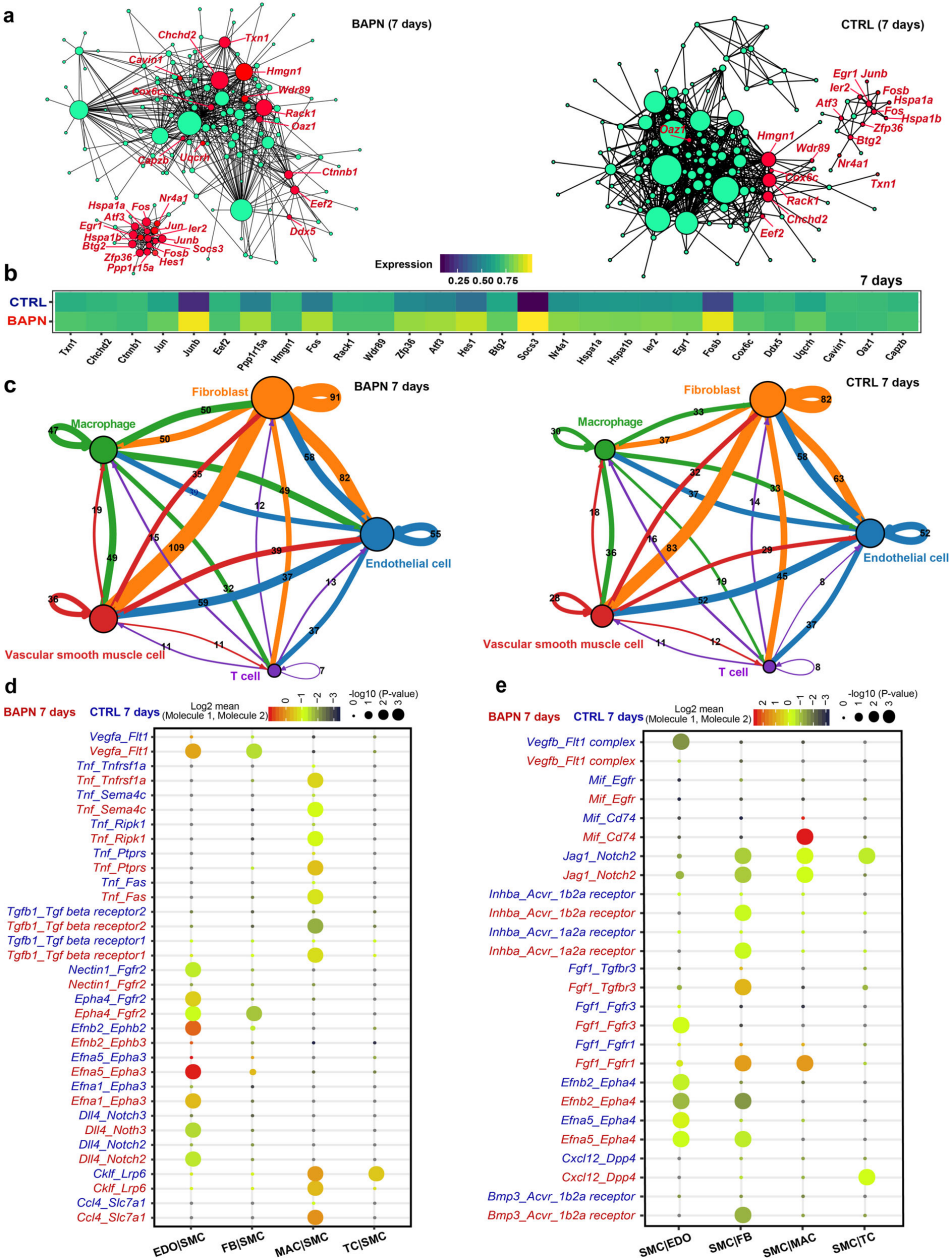
Gene regulatory network of aortic SMCs and inter-lineage communication analysis of the aortic tissues at the early stage of TAAD. **a** Comparative analysis of the gene regulatory networks of SMCs between BAPN (left panel) and CTRL (right panel) reveals dysregulated genes in the early stage of TAAD (7 days after BAPN administration). The node size reflects degree centrality. The top 28 genes ranked by delta degree are colored in red. **b** The average expression of the top dysregulated genes in SMCs of BAPN and CTRL. **c** Cell–cell communication networks in the aortic tissues of BAPN (left panel) and CTRL (right panel). The node size reflects the total number of communications for each lineage. The edge color indicates that the ligands are broadcast by the cell lineage in the same color. The line thickness is proportional to the number of broadcast ligands. **d** The ligand–receptor pairs with significant changes in specificity between any one of the non-SMC lineages and SMCs in BAPN vs CTRL at the early stage of TAAD. SMCs express receptors and receive ligand signals from other lineages. **e** The representative ligand–receptor pairs with significant changes in specificity between SMCs and the other lineages in BAPN vs CTRL at the early stage of TAAD. SMCs express ligands and broadcast ligand signals for other lineages. In **d** and **e**, the dot size reflects the *P* value of the permutation tests for lineage-specificity, and the dot color denotes the mean of the average ligand–receptor expression in the interacting lineages. CTRL, control; EDO, endothelial cell; FB, fibroblast; MAC, macrophage; SMC, smooth muscle cell; TC, T cell; B cells are not considered due to too few cells.

To further identify key molecules of SMCs at the early stage of pathogenesis, gene regulatory networks were constructed from single-cell data using a novel method implemented in bigScale2^27^, which allowed the quantification of the biological importance of genes. Fig 4a shows the regulatory networks constructed for aortic SMCs from the BAPN and the control groups after 7 days of administration. Comparative analysis between the two networks revealed a list of genes that were significantly upregulated in the BAPN group in terms of degree centrality (the number of edges afferent to a given node; Supplementary Table S4). Increased levels of thioredoxin, an antioxidant enzyme encoded by *Txn1*, have been observed in oxidative stress-associated cardiovascular diseases^28^. We found that *Txn1* ranked first in the list and its expression was upregulated in BAPN (Fig. 4b). A recent study reported that SMC-specific deletion of *Socs3* could induce a moderate pro-inflammatory response, thereby ameliorating aortic dissection^29^. The importance of *Socs3* in the regulatory network was found to be greatly increased, and its expression was significantly upregulated in BAPN. The mechanical stress-responsive transcription factor *Egr1* has recently been implicated in the pathogenesis of TAAD^30^. *Egr1*, upregulated in BAPN, was prioritized to be a potentially key regulator according to our network analysis (Fig. 4a, b). *Atf3*, a transcription factor activated by Toll-like receptor signaling, has been identified as a negative regulator of inflammation^31^. Our analysis showed that *Atf3* was a potentially key regulator in the regulatory network of diseased SMCs, and its expression was upregulated in BAPN (Fig. 4a, b). In addition, other stress-responsive regulators that have been suggested to be activated in SMCs of TAAD^14^, including *Jun*, *Junb*, *Fos*, *Nr4a1*, and *Fosb*, were also identified. We also identified genes that were dysregulated in the GRNs after 14 days or 21 days of BAPN administration (Supplementary Fig. S5 and Table S4). Taken together, our analysis highlights the cellular response to oxidative and mechanical stress in SMCs at the early stage of TAAD.

### Cell–cell communication analysis unraveled alterations in receptor–ligand interactions between SMCs and other lineages during the development of TAAD

To identify the alterations in receptor–ligand interactions between SMCs and other lineages at the early stage of TAAD, we performed cell–cell communication analysis using CellPhoneDB^32^, which provides a statistical framework for predicting enriched interactions between two cell types from single-cell transcriptomics data. Compared with the control, the total number of interactions was increased after 7 days (Fig. 4c), 14 days (Supplementary Fig. S6a), and 21 days (Supplementary Fig. S7a) of BAPN administration, indicating enhanced intercellular communications in diseased conditions. Thereafter, the specific receptor–ligand interactions altered between SMCs and other lineages were identified (Fig. 4d and Supplementary Figs. S6b, S7b). Notably, several interactions between macrophages and SMCs became significantly more specific in BAPN compared with healthy conditions (permutation test *P* value < 0.05; Supplementary Table S5). For example, the interactions of tumor necrosis factor-α (TNF-α, encoded by *Tnf*), a pro-inflammatory cytokine secreted by macrophages, and its corresponding receptors (e.g., tumor necrosis factor receptor type 1 encoded by *Tnfrsf1a*) became significantly more specific in BAPN (Fig. 4d). This suggests that macrophages might contribute to TNF-mediated SMC apoptosis at the early stage of TAAD^33^. In addition, macrophages secreted transforming growth factor beta-1 (encoded by *Tgfb1*). Tgfb1–Tgf beta-receptor interactions between macrophages and SMCs became significantly more specific, thereby reflecting the contribution of macrophages to the activation of TGF beta signaling of SMCs in TAAD^34^. Besides, the changes in ligand signals broadcasted by SMCs and received by other lineages were also examined (Fig. 4e and Supplementary Figs. S6c, S7c). Notably, SMCs might exert the impacts on the physiological changes of other lineages including fibroblasts, macrophages, and endothelial cells via paracrine signals of fibroblast growth factor 1 (encoded by *Fgf1*) at the early stage of TAAD (Fig. 4e).

### The heterogeneity of macrophages during the development of TAAD

**Fig. 5:**
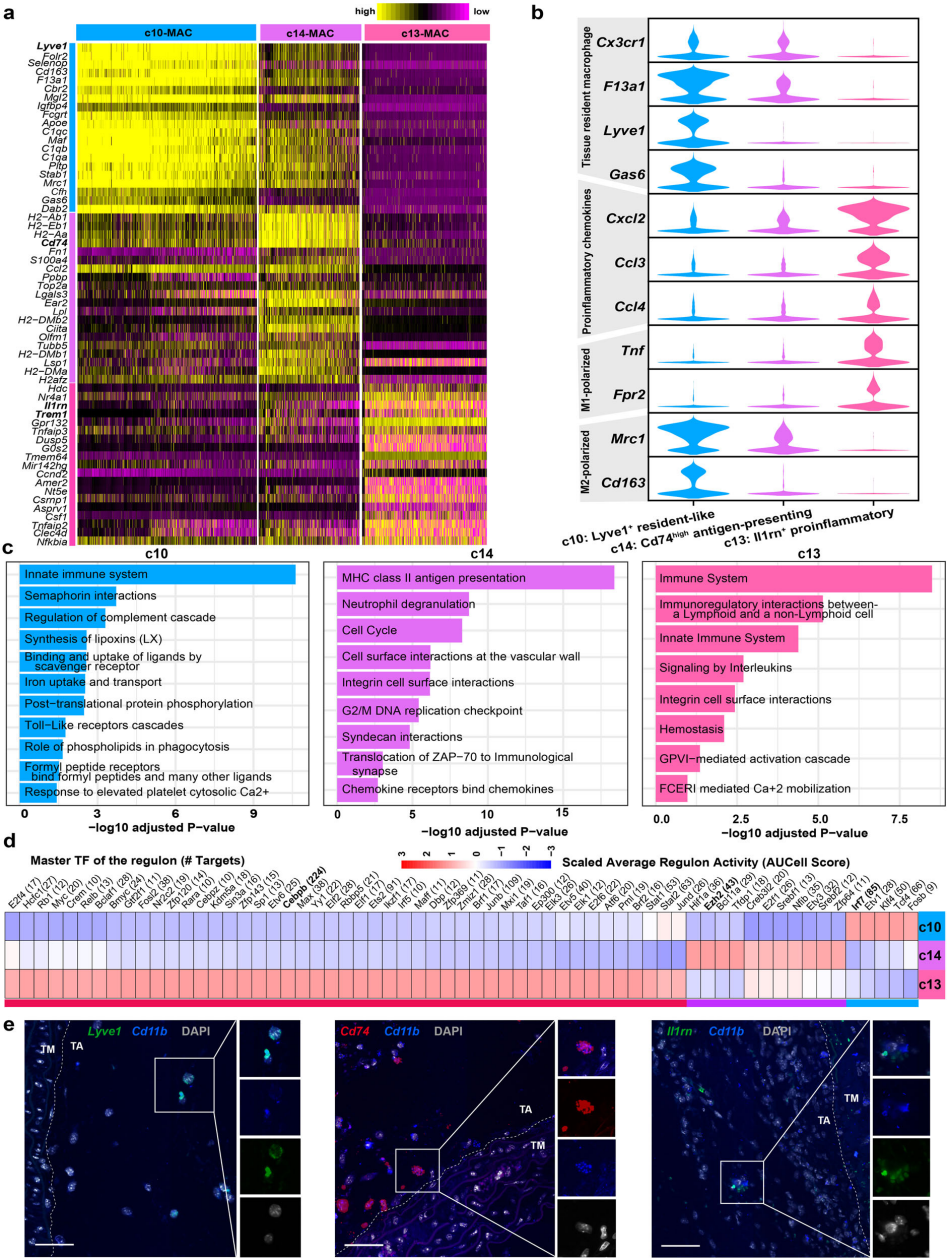
The heterogeneity of macrophages during the development of TAAD. **a** Heatmap showing distinct expression profiles of the three macrophage subpopulations. **b** The expression of markers for pro-inflammatory chemokines, M1-polarized, M2-polarized, and tissue-resident macrophages in the three subpopulations. **c** GO terms enriched in the molecular signatures of each subpopulation. Adjusted *P* value of hypergeometric test <0.05. **d** Subpopulation-specific regulons of each subpopulation revealed by SCENIC analysis. **e** smFISH results showing the spatial distribution of the three macrophage subpopulations in mouse aorta. Scale bars, 50 μm. TA, tunica adventitia; TM, tunica media.

Next, the heterogeneity of macrophages during the development of TAAD was molecularly characterized. The three subpopulations of macrophages exhibited distinct expression patterns (Fig. 5a). Cluster c10 (*Lyve1*^+^) expressed genes related to tissue-resident macrophages including *Cx3cr1*, *F13a1*, *Lyve1*, and *Gas6* (Fig. 5b), thus representing resident-like macrophages as previously proposed^35^. Cluster c14 (*Cd74*^high^) expressed high levels of genes implicated in antigen presentation, such as *Cd74* and MHC II genes (Fig. 5a). Accordingly, the molecular signature of c14 was enriched for GO terms such as “MHC class II antigen presentation” and “cell cycle” (Fig. 5c). Moreover, c14 was phenotypically close to c10 with moderate expression of resident- (e.g., *F13a1*) or M2-polarized macrophage markers (e.g., *Mrc1*)^36^. Collectively, c14 represented a specialized, activated state of macrophages with properties of antigen presentation and proliferation. Cluster c13 (*Il1rn*^+^) expressed high levels of pro-inflammatory chemokines (e.g., *Cxcl2* and *Ccl3*) and M1-polarized macrophage markers (e.g., *Tnf* and *Fpr2*)^37^, thus representing tissue-infiltrated pro-inflammatory macrophages. Furthermore, subpopulation-specific regulons were identified via SCENIC analysis^38^. As shown in Fig. 5d and Supplementary Table S6, large regulons with many target genes were identified for each subpopulation (e.g., *Cebpb* for c13, *Ezh2* for c14, and *Irf7* for c10), which might represent key regulators for maintenance of the specific cellular states.

Then, the presence of the three macrophage subpopulations was confirmed in mouse aorta (all localized in tunica adventitia) using smFISH (Fig. 5e). Also, the presence of the three macrophage subpopulations was confirmed in human samples (Supplementary Fig. S8). Both *LYVE1*^+^ resident-like and *IL1RN*^+^ pro-inflammatory macrophage subpopulations were localized mainly in tunica adventitia. Notably, the *CD74* ^high^ subpopulation was found in all three layers of the aorta in human patients.

### The pro-inflammatory macrophage subpopulation is a predominant source of most detrimental molecules during the development of TAAD

**Fig. 6:**
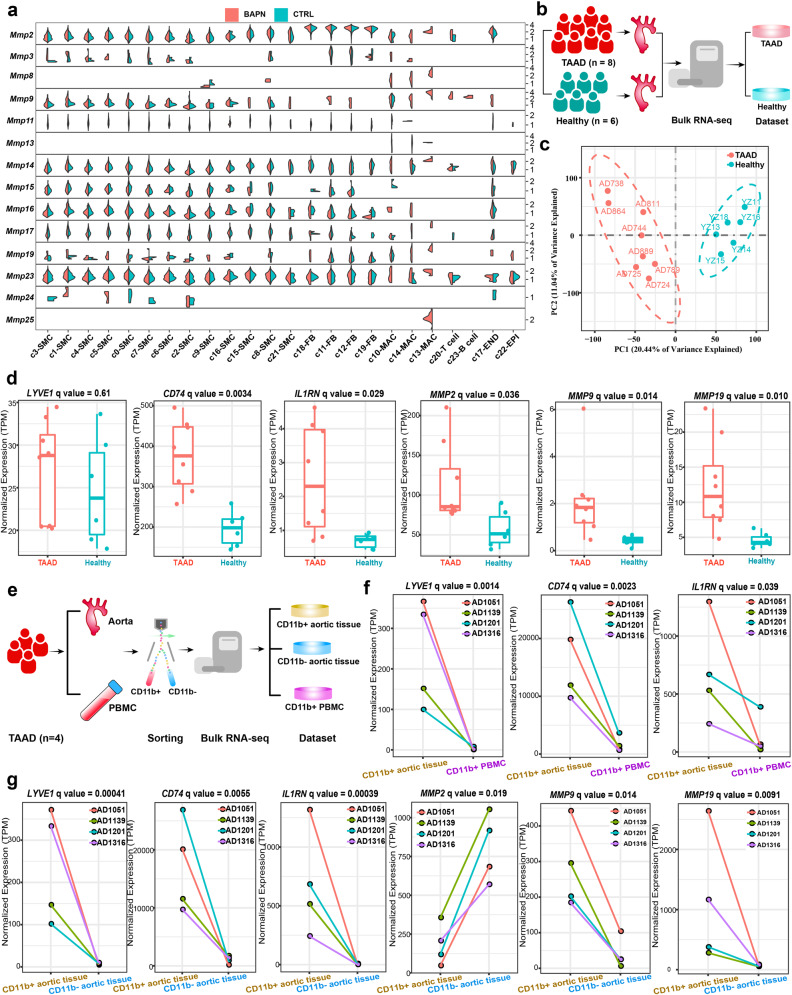
The pro-inflammatory macrophage subpopulation is a predominant source of most detrimental molecules in the development of TAAD. **a** Split violin plot showing the expression of genes encoding MMP family proteins in the mouse aorta of the BAPN and CTRL groups. scRNA-seq data from all three time points were considered. Log2 transformation was performed on the y-axis. CTRL control. **b** Experimental procedure for bulk RNA-seq of the aorta tissues from TAAD patients (*n* = 8) and healthy donors (*n* = 6). **c** Principal component analysis showing the separation of the TAAD from the healthy group. **d** The expression of the markers of the three macrophage subpopulations and three MMP genes in aorta tissues from TAAD patients and healthy donors. The statistical threshold of the differential expression test was set to be a *q* value < 0.05. **e** Experimental procedure for bulk RNA-seq of CD11b^+^ aortic cells, CD11b^−^ aortic cells, and CD11b^+^ PBMCs from TAAD patients (*n* = 4). **f** The expression of the markers of the three macrophage subpopulations in CD11b^+^ aortic tissues and corresponding CD11b^+^ PBMCs. **g** The expression of the markers of the three macrophage subpopulations and three MMP genes in CD11b^+^ and CD11b^−^ aortic cells from TAAD patients and healthy donors. The statistical threshold of the differential expression test was set to be a *q* value < 0.05.

MMP family proteins, including MMP2 and MMP9, have been suggested to play vital roles in the maladaptive degradation and remodeling of ECM in TAAD^4^. We examined the expression of MMP genes (Fig. 6a). Notably, the expression levels of most MMP genes were remarkably higher in macrophages, especially in the pro-inflammatory subpopulation c13. Moreover, the expression levels of MMP genes in macrophages were generally higher in BAPN compared with the control. Besides MMPs, inflammatory cytokines represent another class of detrimental molecules that significantly contribute to the pathogenesis of TAAD, such as IL1β^39^ and TNF-α^40^. We found that *Il1b* and *Tnf* were predominantly expressed by c13 (Supplementary Fig. S9). These findings suggest that the pro-inflammatory subpopulation of macrophages is a predominant source of most detrimental molecules in the development of TAAD.

To verify our findings in human patients, bulk RNA-seq of thoracic aortic tissues from TAAD patients and healthy donors was conducted (Fig. 6b and Supplementary Table S7). Based on the transcriptome, the TAAD group could be separated from the healthy group (Fig. 6c). A total of 540 differentially expressed genes were identified (Supplementary Fig. S10a and Table S8). Representative GO terms that were enriched in the upregulated genes in TAAD were “extracellular matrix organization”, “leukocyte activation”, “inflammatory response”, and “cytokine-mediated signaling pathway” (Supplementary Fig. S10b), which reflects a strong inflammatory response in the aorta of TAAD patients. Then, the expression of markers of the three macrophage subpopulations was examined, which could serve as a proxy of the relative proportion in aortic tissues. As shown in Fig. 6d, *CD74* (c14 marker) and *IL1RN* (c13 marker) were significantly upregulated in TAAD compared to healthy controls (*q* value < 0.05), except for *LYVE1* (c10 marker). As expected, *MMP2* and *MMP9*, the two most studied MMP genes in TAAD, were significantly upregulated in TAAD (Fig. 6d). Also, *MMP19* was significantly upregulated in TAAD, which is consistent with a previous report^41^.

To further explore the properties of macrophages in TAAD, we performed bulk RNA-seq of aortic tissue-derived and peripheral blood mononuclear cells (PBMCs)-derived CD11b^+^ macrophages/monocytes, as well as aortic tissue-derived CD11b^–^ cells (Fig. 6e). Differential expression analysis between aortic tissue-derived and PBMC-derived CD11b^+^ macrophages/monocytes showed that the expression of the markers of the three macrophage subpopulations was significantly higher in aortic macrophages (Fig. 6f and Supplementary Table S9). This suggested that the three macrophages represented specialized macrophages in the tissue microenvironment and phenotypically differed from the macrophages/monocytes in the peripheral circulation. Differential expression analysis between aortic tissue-derived CD11b^+^ macrophages and CD11b^–^ non-macrophage cells further confirmed that the three markers were expressed specifically in macrophages (Fig. 6g and Supplementary Table S10). In line with the results that fibroblasts expressed high levels of *Mmp2* in mice (Fig. 6a), the expression of *MMP2* was significantly higher in CD11b^–^ cells (Fig. 6g), indicating that macrophages are not the major source of *MMP2*. Notably, the expression of *MMP9* and *MMP19* were significantly higher in aortic tissue-derived CD11b^+^ macrophages, indicating that macrophages are the predominant source of these two MMPs.

Together, our results thus suggest that in both mice and humans, aortic tissue-specific macrophages, particularly the *Il1rn*^+^ pro-inflammatory macrophage subpopulation, represent the predominant source of most detrimental molecules, including MMPs and inflammatory cytokines, which promote the development of TAAD.

### Suppression of macrophage accumulation in the aorta with an inhibitor Ki20227 could significantly decrease the incidence of TAAD and aortic rupture in mice

To evaluate the effects of macrophage blockade on the development of TAAD in vivo, mice were administrated a highly selective macrophage colony-stimulating factor (M-CSF) receptor tyrosine kinase inhibitor Ki20227^42^ starting from the first or the 14th day of BAPN administration to deplete the aortic macrophages (Supplementary Fig. S11a). The incidence of TAAD and aortic rupture in mice was significantly decreased with Ki20227 treatment (Supplementary Fig. S11b; Pearson’s *χ*^2^ test, *P* < 0.05). The integrity of the aortic wall was improved in TAAD mice treated with Ki20227 (Supplementary Fig. S11c). The degradation of elastin fibers was reduced by Ki20227 treatment (elastin degradation grade 0–1) compared with the untreated group (elastin degradation grade 3, Supplementary Fig. S11d). Then, the relative expression changes of genes related to the progression of TAAD in response to Ki20227 treatment were examined via quantitative real-time polymerase chain reaction (qPCR, Supplementary Fig. S11e and Table S11). Indeed, the number of aortic macrophages was significantly reduced by Ki20227, as evidenced by the decreased expression of macrophage markers *Cd11b* and *Cd68*. Furthermore, all the three subpopulations of macrophages were depleted, as demonstrated by the decreased expression of three subpopulation-specific markers (*Lyve1*, *Cd74*, and *Il1rn*). The expression of receptor genes implicated in pro-inflammatory cytokine signaling pathways, including *Il1r1* and *Tnfr1*, was significantly downregulated, whereas the expression of *Il4* encoding a cytokine associated with M2-like inflammation was upregulated. These results suggest that the inflammation in the aorta was attenuated following Ki20227 treatment. Additionally, the collagen genes (e.g., *Col1a1* and *Col3a1*) and MMP genes (e.g., *Mmp2* and *Mmp19*) were significantly downregulated by the Ki20227 treatment, indicating the positive effects of Ki20227 on alleviating the adverse ECM remodeling. Together, suppression of the M-CSF-dependent macrophage accumulation in the aorta could decrease the incidence of TAAD and aortic rupture in mice.

### Pharmacological blockade of Trem1 that is predominately expressed by the macrophage subpopulation c13 using mLR12 decreases aortic rupture rate

**Fig. 7:**
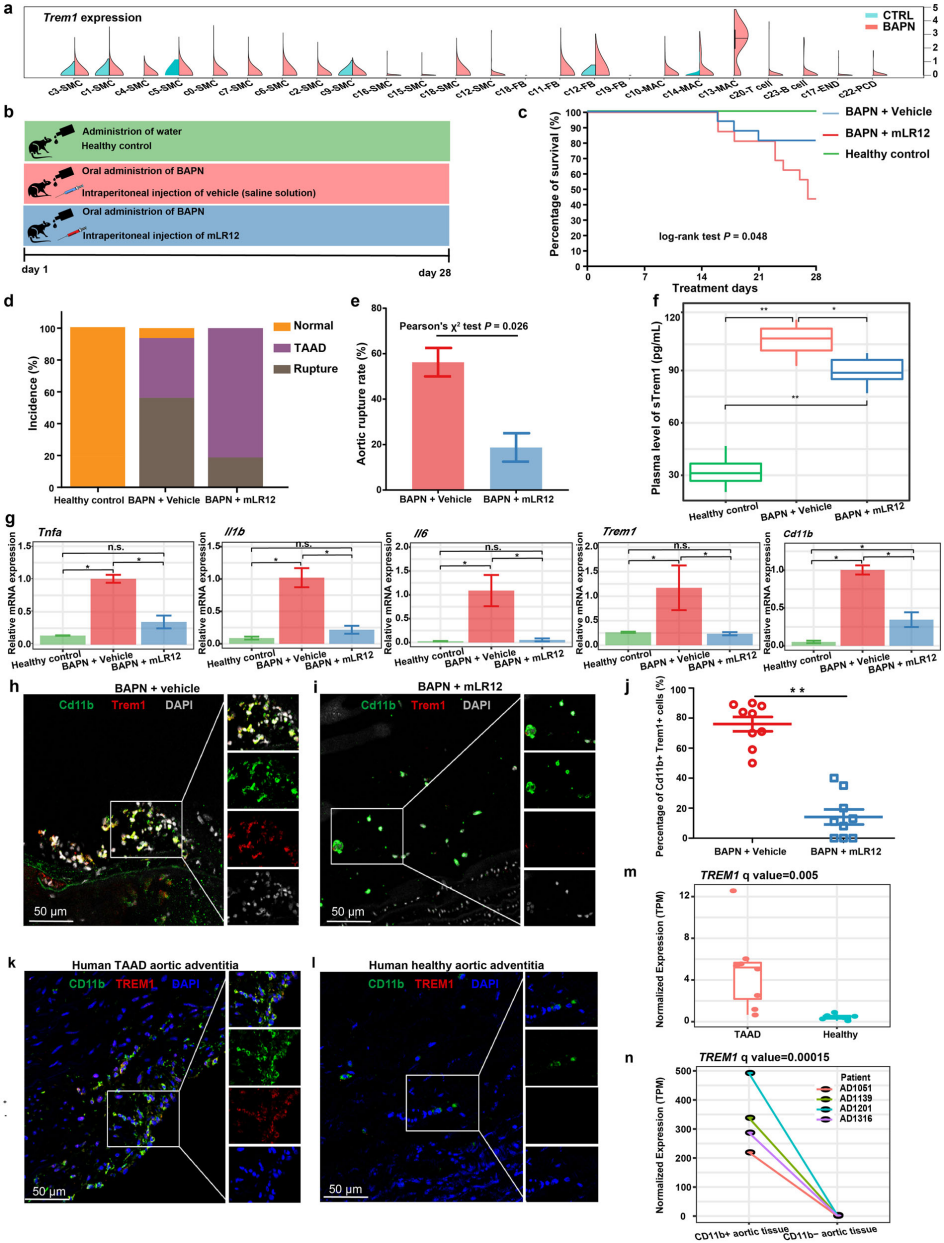
Pharmacological blockade of Trem1 using mLR12 decreases the aortic rupture rate. **a** *Trem1* was highly expressed in the macrophage subpopulation. **b** Schematic illustration of the treatment procedure. Two biological replicates (*n* = 8 for each replicate) were performed for each group. All measurements were performed after 28 days of treatment. **c** Survival curves of mice treated with mLR12 or vehicle (saline solution). **d** Summary of the normal, TAAD, and rupture rates in each group. **e** mLR12 treatment could significantly decrease the aortic rupture rate. **f** Quantification of plasma levels of sTREM1. *n* = 6 for each group. **g** Quantification of the mRNA expression of *Tnfa*, *Il1b*, *Il6*, *Trem1*, and *Cd11b* in the aorta by qPCR (*n* = 3). **h** Immunofluorescence staining for Cd11b^+^Trem1^+^ cells in mouse aorta of the mLR12 treatment group. **i** Immunofluorescence staining for Cd11b^+^Trem1^+^ cells in mouse aorta of the BAPN + vehicle group. **j** The percentage of the Cd11b^+^Trem1^+^ cells in each sample was calculated based on the immunofluorescence staining. Wilcoxon rank-sum test, *P* = 0.0004. *n* = 9 for each group. **k** Immunofluorescence staining for CD11b^+^TREM1^+^ cells in aortic adventitia of human TAAD patients. **l** Immunofluorescence staining for CD11b^+^TREM1^+^ cells in aortic adventitia of healthy donors. **m** The expression of *TREM1* in aortic tissues of human TAAD patients (*n* = 8) and healthy donors (*n* = 6) measured by bulk RNA-seq. **n** The expression of *TREM1* in CD11b^+^ and CD11b^−^ cells sorted from the aortic tissues of TAAD patients (*n* = 4) measured by bulk RNA-seq. In **f** and **g**, all values are means ± SEM. *P < 0.05, **P < 0.01, n.s. not significant, Kruskal–Wallis test followed by multiple pairwise comparisons between groups.

We noted that *Trem1* was highly expressed in the macrophage subpopulation c13 (Fig. 7a), which encodes an immune receptor that amplifies the inflammatory response and regulates myeloid recruitment into inflammatory site^43^. LR12 peptide, a decoy receptor that effectively blocks Trem1 engagement, has recently been proven to be effective in mitigating the development of sterile inflammation-related diseases such as atherosclerosis^44^ and abdominal aortic aneurysm^43^. We thus evaluated the effects of pharmacological blockade of Trem1 using murine LR12 (mLR12) in BAPN-induced TAAD models (Fig. 7b). Survival analysis showed that the mLR12 treatment had better survival compared with the BAPN + vehicle group (Fig. 7c, log-rank test, *P* = 0.048). mLR12 treatment could significantly decrease the incidence of death due to aortic rupture (Fig. 7d, e; Pearson’s *χ*^2^ test, test, *P* = 0.026). Plasma levels of the secreted soluble form of Trem1 (sTrem1) in the mLR12 treatment group were significantly lower than that in the BAPN + vehicle (Fig. 7f; Kruskal–Wallis test following by post hoc tests, *P* < 0.05). The expression of genes encoding pro-inflammatory cytokines such as *Tnfa*, *Il1b*, and *Il6* was significantly decreased in the mLR12 treatment group compared with the vehicle group (Fig. 7g; Kruskal–Wallis test followed by post hoc tests, *P* < 0.05), reflecting an attenuated inflammatory response. No significant difference in the expression of these cytokine genes was observed between the mLR12 treatment group and the healthy control group (Fig. 7g). The expression of *Trem1* and *Cd11b* was also decreased (Fig. 7g; Kruskal–Wallis test followed by post hoc tests, *P* < 0.05). Immunofluorescence staining showed a significantly reduced number of Cd11b^+^Trem1^+^ cells in the aortic tissue of the mLR12 treatment group compared with the BAPN + vehicle group (Wilcoxon rank-sum test, *P* = 0.0004; Fig. 7h–j and Supplementary Figs. S12a, S13), reflecting a reduced infiltration of pro-inflammatory macrophages. In addition, immunofluorescence staining showed a significant expansion of CD11b^+^ TREM1^+^ cells in aortic adventitia of TAAD patients compared with that of healthy donors (Fig. 7k, l and Supplementary Fig. S12b). Consistently, the expression of TREM1 was significantly higher in the aortic tissues from TAAD patients (Fig. 7m, *q* value = 0.005). TREM1 was predominately expressed in CD11b^+^ cells sorted from the aortic tissues of TAAD patients (Fig. 7n, *q* value = 0.00015). Together, our results suggest that targeting the macrophage subpopulation c13 via blockade of TREM1 may constitute a novel and promising treatment to slow the progression and prevent aortic rupture in TAAD.

## Discussion

Compared with AAAD of which inflammation is a cardinal feature, investigations on the role of inflammation in TAAD are much less extensive^4^. Accumulating evidence shows increased infiltration of immune cells, particularly macrophages and T cells, in the aortic tissues of TAAD^14,45^. Nevertheless, the precise roles of inflammation and immune cells in TAAD remain elusive. Inflammatory cytokines and immune cell-related MMPs are representative detrimental molecules for the development of TAAD. The contributions of a few inflammatory cytokines and MMPs in TAAD have been established by a genetic deletion in mice. For instance, *Il1b* knockout could decrease TAA formation and progression^39^. *Mmp9* gene deletion could attenuate TAA formation^46^. However, the expression patterns of these detrimental molecules during the development of TAAD have not been explored at the single-cell level. In this study, we provided multiple lines of evidence demonstrating the contribution of inflammation during the development of TAAD, which highlights the importance of anti-inflammation therapy in TAAD. First, we identified three macrophage subpopulations whose proportion changes were associated with the progression of TAAD in mice (Fig. 2). Bulk RNA-seq of the thoracic aortic tissues of human patients revealed a strong inflammatory response in the aorta (Supplementary Fig. S10b). The three macrophage subpopulations were confirmed in human samples (Supplementary Fig. S8), and the antigen-presenting subpopulation and pro-inflammatory subpopulation were significantly expanded in the aortic tissues from human patients (Fig. 6d). Second, cell–cell communication analysis revealed that macrophages might promote TNF-mediated SMC apoptosis and the activation of TGF beta signaling in SMCs at the early stage of TAAD (Fig. 4d). Third, for the first time, we demonstrated that in both mice and humans, the *Il1rn* + pro-inflammatory macrophage subpopulation c13 represents the predominant source of most detrimental molecules, including MMPs and inflammatory cytokines, which contribute to the development of TAAD (Fig. 6). Fourth, suppression of macrophage accumulation in the aorta with an inhibitor Ki20227 could mitigate the progression of TAAD in vivo (Supplementary Fig. S11), suggesting the central role of macrophage-orchestrated inflammation responses in the pathogenesis of TAAD. Lastly, targeting the macrophage subpopulation c13 via blockade of Trem1 using mLR12 could decrease the aortic rupture rate (Fig. 7). Instead of inhibiting all macrophages, targeting the pro-inflammatory subpopulation c13 may constitute a promising medical treatment.

Macrophages represent the most abundant immune cells and the hub of inflammation in the aortic wall^47^. They are functionally heterogeneous, which can be pro-inflammatory or anti-inflammatory. In response to environmental cues, macrophages display remarkable plasticity. Another level of heterogeneity of tissue macrophages is embryonic origin: tissue macrophages can be derived from erythro-myeloid progenitors (EMPs) of the yolk sac or bone marrow (BM) via monocyte trans-differentiation. The precise roles of aortic macrophage subpopulations have not been elucidated in TAAD. Through unbiased clustering of single-cell transcriptomes, we identified three macrophage subpopulations in the thoracic aortic tissues: *Lyve1*^+^ resident-like macrophages (cluster c10), *Cd74*^high^ antigen-presenting macrophages (cluster c14), and *Il1rn*^+^ tissue-infiltrated pro-inflammatory macrophages (cluster c13). We noted that the three subpopulations resembled the three aortic macrophage subpopulations that were recently reported by combining lineage tracing and single-cell analysis^48^, i.e., homeostatic and anti-inflammatory resident macrophages (*Lyve1*^+^; mostly EMP-derived), antigen-presenting macrophages (*Cd74*^+^; both EMP- and BM-derived) as well as inflammatory macrophages (*Il1b*^+^; BM-derived). Based on the information above, the changes of macrophage subpopulations during the development of TAAD can be associated with an embryonic origin. The expansion of resident-like macrophages (cluster c10) was mediated by local proliferation of EMP-derived cells, while the significant expansion of pro-inflammatory macrophages (cluster c13) was mediated by recruitment of BM-derived monocytes. While *TREM1* has previously been known to be constitutively expressed by neutrophils and monocytes/macrophages^43^, we found that *Trem1* was predominately expressed by a specific aortic tissue-infiltrated macrophage subpopulation c13 that was differentiated from BM-derived monocytes in TAAD mouse models. TREM1 plays a crucial role in the amplification of the inflammatory response and also orchestrates myeloid recruitment into inflammatory sites^43^. The reduced expression of Trem1 and Cd11b (macrophage marker) in the aortic tissues following the treatment of mLR12 (Fig. 7) suggests a decreased macrophage content (especially the c13 pro-inflammatory macrophages), which may result from an attenuated inflammatory response and a reduction of the classical monocyte trafficking to the aortic tissues.

Thoracic aortic aneurysms are mostly asymptomatic before the onset of fatal complications including rupture. Understanding the cellular and molecular events at the onset of TAAD is crucial for designing medical therapies for prevention or treatment. Therefore, we particularly focused on the mechanisms of TAAD onset based on the early stage of mouse models (after 7 days of BAPN induction). Regarding the onset of sporadic TAAD, a so-called “double-face signal pathway model” was previously proposed^49^. This model highlights the central role of the TLR4 signaling pathway, a classic receptor signaling pathway of innate immunity that has recently been implicated in the pathogenesis of a variety of noninfectious diseases such as atherosclerosis^50^ and abdominal aorta aneurysm^51^. In this model, environmental stimuli including mechanical stress induced by hypertension lead to the accumulation of reactive oxygen species (ROS) and the overactivation of stress-responsive pathways, which further causes cell damage and tissue injury. The damaged cells could release a plethora of endogenous molecules called damage-associated molecular patterns (DAMPs), such as heat shock proteins (HSPs) and low molecular hyaluronic acid. High levels of DAMPs could be recognized by TLR4, which results in sustained activation of the TLR4 signaling pathway that evokes chronic inflammation and immune cell infiltration^52^. Consequently, aortic cells exhibit a so-called senescence-associated secretory phenotype (SASP), characterized by the release of pro-inflammatory mediators and MMPs. It acts as a vicious cycle to further induce cell damage and DAMP accumulation. Ultimately, it causes medial degradation and the onset of TAAD. Despite this model being mostly supported by genetic evidence^49^, our study provides supportive evidence to this model based on single-cell analysis. Through GSEA, we found that the pathways “oxidative stress-induced senescence”, “intrinsic apoptotic signaling pathway in response to ER stress”, “TOLL-like receptor 4 TLR4 cascade”, and “senescence-associated secretory phenotype” were significantly upregulated in SMCs at the early stage of TAAD (Fig. 3 and Supplementary Fig. S3). Furthermore, gene regulatory network analysis revealed a list of key molecules expressed in SMCs at the early stage (Fig. 4a, b), including stress-responsive regulators, such as *Egr1*, a mechanical stress-responsive transcription factor^30^, and *Atf3*, an adaptive response transcription factor activated by TLR signaling^31^. Therefore, our study highlights the excessive activation of stress-responsive pathways and TLR signaling in SMCs at the onset of TAAD. It is also noteworthy that TREM1 signaling in macrophages partners with TLR signaling^53^. Medical therapies targeting DAMPs and TLR signaling may also serve as a promising way to mitigate the progression of TAAD.

In summary, we present a comprehensive analysis of the dynamics of cellular and molecular changes during the development of TAAD. Our study pinpoints the pro-inflammatory macrophage subpopulation (c13, *Il1rn*^+^/*Trem1*^+^) as the predominant source of most detrimental molecules for TAAD. Furthermore, we demonstrated that targeting the macrophage subpopulation c13 via blockade of Trem1 using mLR12 could decrease the aortic rupture rate, which may constitute a novel and promising medical treatment to mitigate the progression and prevent aortic rupture in TAAD. In addition, our findings highlight the contribution of excessive activation of stress-responsive and TLR signaling pathways in aortic SMCs at the onset of TAAD and support the previously proposed “double-face signal pathway model” regarding the onset of sporadic TAAD. Overall, our study highlights the importance of anti-inflammation therapy in TAAD. Our dataset constitutes a valuable resource for further interrogating the pathogenesis mechanisms of TAAD.

## Materials and methods

### Ethics statement

Animal experiments and the use of human samples were approved by the ethics committee of Fuwai Hospital (FW-2019-0008). All applicable institutional and/or national guidelines for the care and use of animals were followed. Written informed consent was received from each patient.

### BAPN-induced TAAD mouse model

The TAAD mouse model was induced based on previously described methods with modifications^25^. Briefly, 3-week-old male C57BL/10 mice were daily administrated a solution of BAPN (Sigma-Aldrich, USA) dissolved in drinking water (0.5 g/kg weight) for 28 days. The ascending aorta and aortic arch were collected at three time points during the induction, i.e., after 7, 14, and 21 days of BAPN administration. An autopsy was performed immediately to confirm whether they died of aortic problems, e.g., aortic rupture, during the induction. As a control, tissue samples were also collected at each time point from mice administrated vehicle (drinking water). To mitigate the effects of individual variance, we pooled the tissues of six mice for each time point in single-cell experiments.

### Animal treatment with Ki20227

Three-week-old male C57BL/10 mice received oral daily administration of BAPN (0.5 g/kg/day) to induce TAAD. To assess the effect of macrophages on the induction, the macrophage inhibitor Ki20227 (DC CHEMICAL, DC7168; 30 mg/kg)^54^ was administrated together with BAPN from the first or the 14th day of induction (*n* = 8). As a control, mice were also treated with vehicle (0.05% methylcellulose) and BAPN (*n* = 8). Three replicates were performed for each treatment. The induction rate of TAAD was accessed on the 28th day of induction.

### Animal aortic tissue dissection

Mice were euthanized by CO_2_ overdose. 75% ethanol was sprayed onto abdominal fur. Then, the chest was opened and the inferior vena cava was snipped. To remove blood cells, the aorta was perfused from the left ventricle with 0.05 mM ethylenediaminetetraacetic acid (EDTA) in Dulbecco’s Phosphate-Buffered Saline (DPBS, Sigma-Aldrich, USA). Subsequently, the aorta was finely dissected under a stereoscope. The adipose tissue adjacent to the adventitia was carefully removed. Finally. the ascending and aortic arch regions of the aorta were obtained and placed in cold Hank’s Balanced Salt Solution (HBSS; Thermo Fisher Scientific, USA), which were immediately dissociated to single cells after the dissection.

### Tissue dissociation and preparation of single-cell suspension

The obtained tissue was cut into vascular rings under a stereoscope. Then, the vascular rings were transferred into EP tubes and were washed with DPBS. For tissue dissociation, Pierce™ Primary Cardiomyocyte Isolation Kit (Thermo Fisher Scientific™, Cat. No. 88281) was used following the manufacturer’s instructions. Afterward, we performed blind shear and bathed it at 37 °C for 25 min. After the digestion process, 400–500 μL DPBS was added to the mixture, pipet-mixed gently, and then filtered with a 40-μm filter to collect the dissociated cells. Thereafter, the mixture was centrifuged for 5 min (4 °C, 400 × *g*). The supernatant was discarded and a 200 μL cold Sample Buffer (BD R added hapsody™, Cat. No. 650000062) was added to the cell mass. Finally, the mixture was pipet-mixed gently to obtain the cell suspension for single-cell experiments.

### Cell capture, scRNA-seq library preparation, and sequencing

For cell capture and library preparation for scRNA-seq, the BD Rhapsody™ system (BD Biosciences, USA) was applied based on the manufacturer’s protocols. In brief, 1 μL Calcein AM (2 mM; Thermo Fisher Scientific, USA, Cat. No. C1430) and 1 μL DRAQ7 (0.3 mM; Thermo Fisher Scientific, USA, Cat. No. 564904) were added to 200 μL cell suspension (1:200 dilution). Then, the mixture was pipet-mixed gently and incubated at 37 °C in dark for 5 min. The cell viability and concentration of the suspension were measured by Hemocytometer Adapter (Cat. No. 633703). The single-cell suspension was then loaded onto a BD Rhapsody cartridge (Cat. No. 400000847) with >200,000 microwells. Thereafter, cell capture beads were loaded into the microwells and washed to make sure that one magnetic bead binds to only one cell in a single microwell. Subsequently, the lysis mix was added and incubated at room temperature (15–25 °C) for 2 min. The cell capture beads were eventually retrieved for subsequent complementary DNA synthesis, exonuclease I digestion, and multiplex PCR-based library construction. Sequencing libraries were prepared using Whole Transcriptome Analysis Index PCR and the PCR product was purified to enrich the 3′ end of the transcripts linked with the cell label and molecular indices. Finally, quality checks of the indexed libraries were performed with a Qubit Fluorometer using the Qubit dsDNA HS Assay. The sequencing of the libraries was conducted on an Illumina NovaSeq 6000 system.

### Bulk RNA-seq of human samples

Thoracic aortic tissue was obtained from 12 TAAD human patients undergoing thoracic aneurysm repair surgery. As a control, thoracic aortic tissue from six donors with cardiomyopathy undergoing cardiac transplantation was also obtained. These tissue samples were then subjected to bulk RNA-seq. Additionally, to examine the transcriptomic characteristics of macrophages/monocytes in TAAD patients, thoracic aortic tissue and peripheral blood from four TAAD patients were collected. CD11b^+^ macrophages/monocytes were sorted with antibody-bound magnetic beads from tissue and PBMCs. CD11b^+^ cells of the tissue, CD11b^–^ cells of the tissue, and CD11b^+^ PBMCs were subjected to bulk RNA-seq. The RNA-seq libraries were constructed and the sequencing data were analyzed using previously described methods^55^. Briefly, according to the manufacturer’s instruction, NEBNext^®^ UltraTM Directional RNA Library Prep Kit for Illumina (NEB, USA) was applied to prepare sequencing libraries. We sequenced the obtained libraries using an Illumina HiSeq X Ten System in a 2 × 150 bp paired-end mode. Following quality control using fastp^56^, sequencing reads were subjected to transcript abundance quantification with Kallisto^57^. Differentially expressed genes between groups were detected using sleuth^58^. The biological significance threshold was set to a fold change of ± 2-fold and the statistical significance cutoff was set to a *q* value of 0.05.

### scRNA-seq data preprocessing

The raw sequencing reads were trimmed to keep the first 75 bases. Then the trimmed reads were quality filtered using fastp^56^. The BD Rhapsody whole transcriptome official analysis pipeline (Version 1.5.1) was applied under the default settings to obtain a cell-gene expression matrix and a quality control report for each sample. The mouse reference genome (version: mm10) was used for read alignment. The expression matrix was imported into Seurat v3.1.0^59,60^ for further preprocessing. We filtered out genes with counts in fewer than three cells to eliminate genes that were most likely discovered due to random noise. We filtered cell outliers (<first quartile – 1.5 interquartile range or >third quartile + 1.5 interquartile range) based on the proportion of mitochondrial genes, the number of expressed genes, and the sum of unique molecular identifier (UMI) counts. To eliminate doublets, cells were filtered out using Scrublet^61^. Furthermore, cells enriched in hemoglobin gene expression were also excluded. We normalized the UMI count sum of each cell to 10,000 and then log-transformed the data. To identify shared cellular states across samples, all the datasets were integrated via canonical correlation analysis (CCA) implemented in Seurat. The G2M phase score, S-phase score, UMI count, and mitochondrial gene proportion were regressed using linear models.

### Dimensional reduction and clustering

Principal component analysis (PCA) was used to reduce the dimensionality of scRNA-seq expression data. A neighborhood graph of the cells was generated based on the first 30 PCA components. Using UMAP, we embedded the neighborhood graph in two-dimensional space^62^. Louvain clustering^63^ was used to cluster the cells (resolution = 1).

### Functional enrichment and differential expression analysis

Using the likelihood-ratio test implemented in the function “FindMarkers” of Seurat, we detected differentially expressed genes between two groups of cells. The significance threshold was set to a log2-fold change >0.25 and adjusted *P* value < 0.05. ClueGO^64^ was used to perform functional enrichment analysis on a gene list with a Bonferroni-corrected *P* value < 0.05.

### Differential proportion analysis

We used a permutation-based statistical test (differential proportion analysis; DPA)^23^ to evaluate whether the change in cell relative proportion was expected by chance compared with the control. All the parameters were set as default. A *P* value < 0.05 was used as the statistical cutoff.

### GSEA

Signal2Noise (the difference of means between BAPN and CTRL scaled by the standard deviation) was used to rank all the genes. The pre-ranked gene list was then loaded to GSEA (version: 4.0.1)^65^. The statistical significance cutoff was set to an FDR *q* value < 0.05. In this analysis, we used the precompiled REACTOME pathways in MSigDB (version: 7.0)^66^. The EnrichmentMap plugin of Cytoscape was applied to visualize the results.

### Gene set-based signature scoring

Using Single-Cell Signature Explorer^67^, gene set-based signature scoring was performed to evaluate the expression activity of a gene set in each cell. We used the precompiled gene sets of REACTOME pathways in MSigDB. Human genes were mapped to their mouse orthologs. The expression activity of a gene set in each sample was represented by the mean of the computed scores across cells in the sample.

### Gene regulatory network analysis

With the function “compute.network” (clustering = “direct”, quantile.p = 0.90) in bigScale2^27^, gene regulatory networks were built from single-cell datasets separately for BAPN and CTRL (the control). Subsequently, using the function “homogenize.networks”, the number of edges was homogenized throughout the networks Lastly, using the function “compare.centrality”, we identified the changes in node centrality (the relative importance of genes in the network) between BAPN and CTRL. We mainly considered the degree centrality that means the number of edges afferent to a given node (gene). The change in network centrality for a given node (gene) was measured by delta degree, which means the difference in the number of edges for the node in the BAPN network vs the CTRLnetwork.

### Cell–cell communication analysis

Using CellPhoneDB 2.0^32^, cell–cell communication was inferred with the single-cell transcriptomic dataset. Briefly, according to the expression of a ligand by one lineage and a receptor by another, potential ligand–receptor interactions were predicted. We only retained receptors and ligands expressed in greater than 10% of the cells in any cell type. To generate a null distribution for each receptor–ligand, we permuted the cell labels randomly 1000 times and computed the means of the average ligand–receptor expression in the interacting cell types. Lastly, for each ligand–receptor pair, we obtained a *P* value for the likelihood of lineage-specificity.

### Subpopulation-specific regulon analysis

Using the R package SCENIC^38^, regulon analysis was carried out to identify the regulators driving the cellular heterogeneity among subpopulations. Briefly, we detected co-expression modules, which consisted of a group of genes co-expressed with regulators. We only retained the modules with significant motif enrichment (referred to as regulons). We scored the activity of each regulon for each cell. The average regulon activity scores in the subpopulation were used to identify subpopulation-specific regulons.

### Single-molecule fluorescent in situ hybridization

Fresh tissue was immediately immersed in 10% neutral buffered formalin and was fixed at room temperature for 16–32 h. After rinsing with running water for 30 min, the tissue was dehydrated in gradient ethanol. After xylene transparent and paraffin embedment, tissue was sliced into 10-μm sections, and the slides were dried at room temperature overnight. Nucleotide probes of the target genes were designed and synthesized by Advanced Cell Diagnostics. smFISH was performed using a highly sensitive RNAscope^®^ technology based on the manufacturer’s instructions. The RNAscope^®^ Multiplex Fluorescent Reagent Kit v2(Cat. No. 323100) was applied for visualizing hybridization signals. Fluorescence images were captured on a confocal microscope (LSM780; Carl Zeiss, Germany) and the Vectra^®^ Polaris™ pathology imaging system (PerkinElmer, USA). The probes used for smFISH included: Hs-LYVE1 (Cat. No. 426911-C3), Hs-CD74 (Cat. No. 477521), Hs-IL1RN (Cat. No. 441601), Hs-ITGAM (Cat. No. 555091-C2), Hs-MYH11 (Cat. No. 444151), Hs-RGS5 (Cat. No. 533421-C4), Mm-Lyve1 (Cat. No. 428451), Mm-Cd74 (Cat. No. 437501-C2), Mm-Il1rn (Cat. No. 495101), Mm-Itgam (Cat. No. 311491-C3), Mm-Rgs5 (Cat. No. 430181), Mm-Myh11 (Cat. No. 316101-C2), negative control (Cat. No. 321831), and positive control (Cat. No. 321811).

### Elastin staining

Elastin staining was performed using the Sigma-Aldrich Elastic Stain kit (Sigma-Aldrich, St. Louis, MO, USA, Cat. No. HT25A-1KT) following the manufacturer’s instructions. Briefly, the aortic tissue sections of mice were deparaffinized and stained with Working Elastic Stain Solution for 10 min. Then, the sections were treated with the Working Ferric Chloride Solution. Subsequently, the sections were immediately rinsed with distilled water and stained in Van Gieson’s Solution for 2 min. Lastly, the sections were dehydrated with ethanol and were made transparent with xylene. Elastin degradation was graded according to the features of elastin fibers: grade 0 (intact fibers with normal physiological curvature), grade 1 (stretched fibers with lost physiological curvature), grade 2 (a few fragmented fibers observed), and grade 3 (severely destructed fibers)^68^.

### Quantitative real-time polymerase chain reaction

Total RNA was extracted from mouse aortic tissue using the TRIzol reagent (Invitrogen, Cat. No. 15596018). To quantify the relative expression of target genes, qPCR with SYBR green as reporter dye was performed using the previously described methods^69^. The housekeeping gene *GAPDH* was used as a control. Three biological replicates were set for each group.

### Animal treatment with Trem1 inhibitory peptide mLR12

To pharmacologically inhibit Trem1, a dodecapeptide, murine LR12 (LQEEDTGEYGCV; mLR12)^53^ was chemically synthesized (SBS Genetech, China), and COOH terminally amidated. Following purification, the purity was above 98%, which was determined by high-performance liquid chromatography and mass spectrometry. The endotoxin was below 0.5 EU/mg. Mice were administrated a solution of BAPN to induce TAAD (0.5 g/kg) and were treated daily by intraperitoneal injection of mLR12 (5 mg/kg) for 28 days. As a control, mice administrated BAPN were intraperitoneally injected daily with saline solution.

### Quantification of plasma level of soluble Trem1

Mouse plasma was collected by centrifugation of blood from eyeballs (2000× *g*, 20 min). The secreted soluble form of Trem1 (sTrem1) was measured using Mouse/Rat TREM1 Quantikine ELISA Kit (R&D Systems, Cat. No. MTRM10) according to the manufacturer’s instructions.

### Immunofluorescence staining

Mouse aortic tissue was fixed with 4% paraformaldehyde, dehydrated, embedded with paraffin, and cut into 3-μm sections. After dewaxing, EDTA PH 9.0 Antigen Retrieval Solution (ZSGB-Bio, Cat. No. ZLI-9069) was used for antigenic repair, and then a blocking solution (ZSGB-Bio, Cat. No. ZLI-9022) was used for sealing at room temperature for 1 h. The primary antibody was treated overnight at 4 °C, and the secondary antibody was treated at room temperature for 40 min. For quantitative analysis, three mice were considered for each group. Three slides were selected from each mouse, and three fields were collected from each slide. Image J was used to count the number of positive cells. The antibodies used included: CD11b (Abcam, Cat. No. ab8878), Trem1 (Bioss, Cat. No. bs-4886R), TREM1(R&D, Cat. No. AF1278), Rat IgG2b (Abcam, Cat. No. ab18536), Goat IgG Control (R&D, Cat. No. AB-108-C), Rabbit IgG Isotype Control (Bioss, Cat. No. bs-0295P), Goat Anti-Rat IgG H&L (Alexa Fluor 488) (Abcam, Cat. No. ab150157), Alexa Fluor 594 Labeled Goat Anti-Rabbit IgG (H + L) (ZSGB-Bio, Cat. No. ZF-0516), and Alexa Fluor 594 Labeled Rabbit Anti-Goat IgG (H + L) (ZSGB-Bio, Cat. No. ZF-0517).

## Supplementary information

Supplementary Figures

Supplementary Table S1

Supplementary Table S2

Supplementary Table S3

Supplementary Table S4

Supplementary Table S5

Supplementary Table S6

Supplementary Table S7

Supplementary Table S8

Supplementary Table S9

Supplementary Table S10

Supplementary Table S11

## Data Availability

The scRNA-seq data have been deposited in Genome Sequence Archive (https://bigd.big.ac.cn/gsa/) and are available through the accession number CRA003013.
