# Supplementary material for: Single-cell RNA sequencing identifies an Il1rn+/Trem1+ macrophage subpopulation as a cellular target for mitigating the progression of thoracic aortic aneurysm and dissection
Source: Cell Discov. 2022 Feb 8;8:11. doi: 10.1038/s41421-021-00362-2 (PMC8821555; doi:10.1038/s41421-021-00362-2)
Supplement: Supplementary file 1 — Supplementary Figures [file 41421_2021_362_MOESM1_ESM.pdf]

Supplementary Information

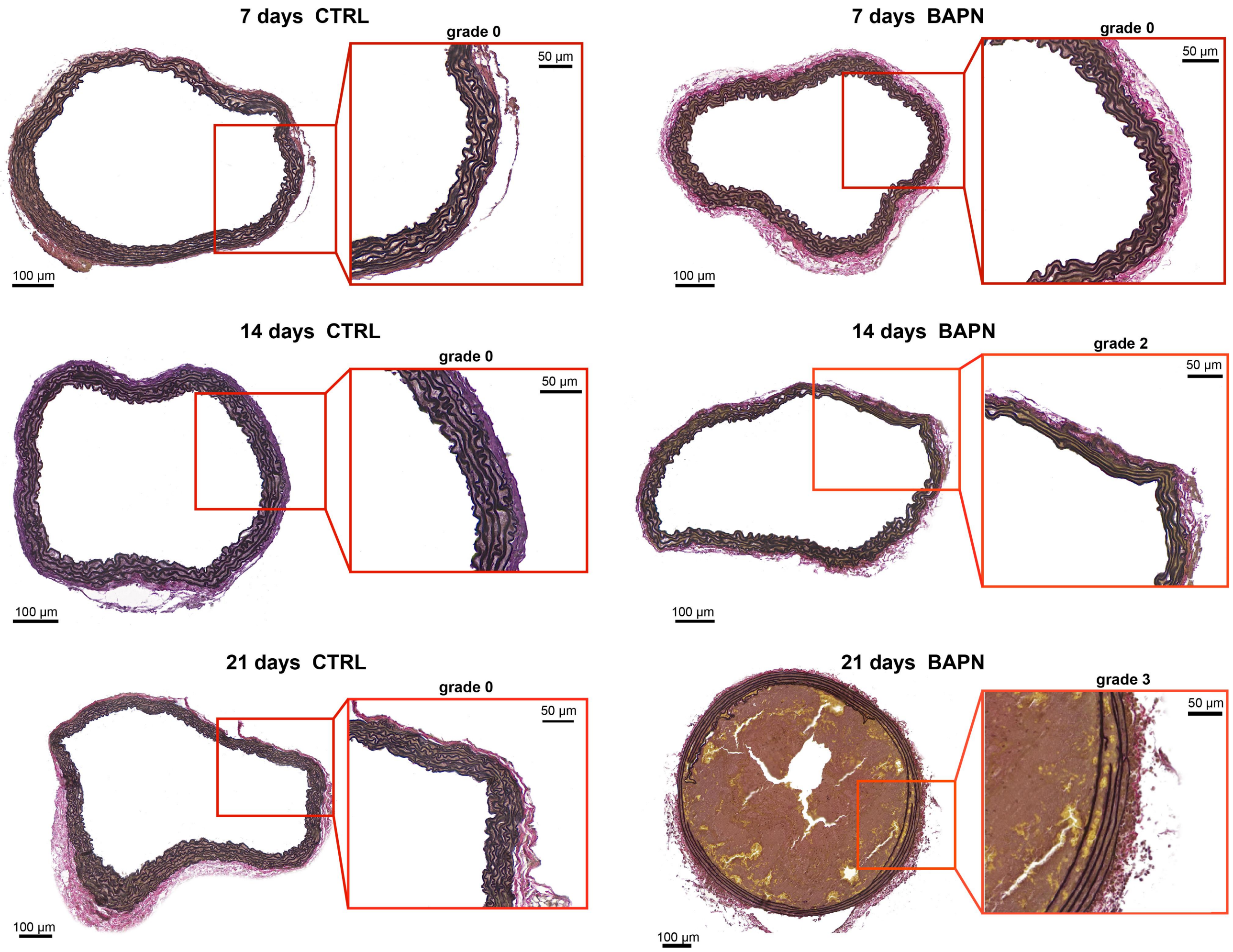

Fig. S1. Elastin staining showing the histopathological changes during the development of TAAD. Elastin degradation was graded according to the features of elastin fibers: grade 0 (intact fibers with normal physiological curvature), grade 1 (stretched fibers with lost physiological curvature), grade 2 (a few fragmented fibers observed), and grade 3 (severely destructed fibers).

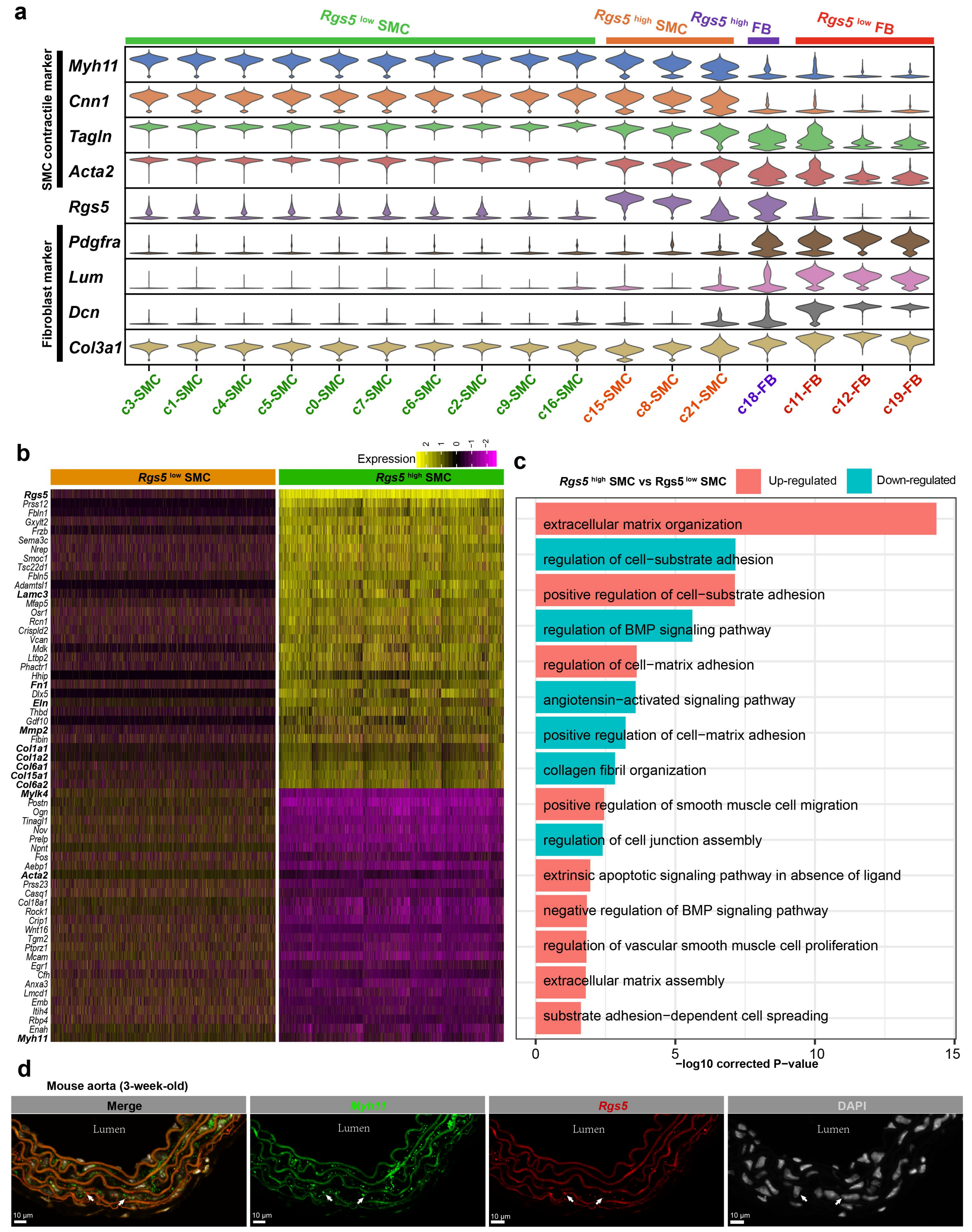

Fig. S2. ***Rgs5* high SMCs are phenotypically modulated SMCs. a** SMCs and fibroblasts could be subdivided into *Rgs5* low SMCs, *Rgs5* high SMCs, *Rgs5* high FBs and *Rgs5* low FBs. **b** Heatmap showing the expression differences of *Rgs5* high and *Rgs5* low SMCs. **c** GO terms enriched in significantly up-regulated or down-regulated genes between *Rgs5* high and *Rgs5* low SMCs. Bonferroni-corrected *P* value of the hypergeometric test < 0.05. **d** smFISH results showing the spatially distribution of *Rgs5* high SMCs. Arrows

indicate the *Myh11*⁺ *Rgs5* high SMCs that are located at the outer layers of the tunica media. 3-week-old healthy mice were used. FB: fibroblast; SMC: smooth muscle cell

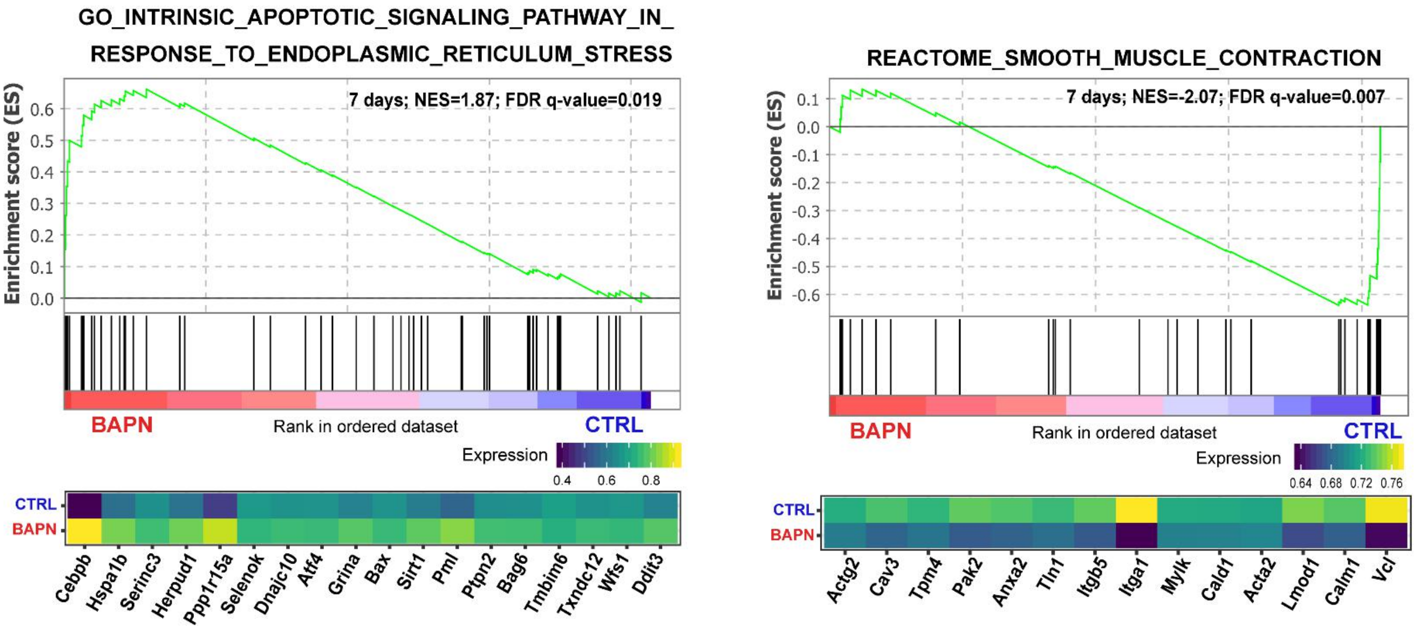

Fig. S3. Enrichment map showing the upregulation of endoplasmic reticulum stress and the downregulation of smooth muscle contraction in BAPN versus control after 7 days of administration. The statistical significance threshold was set to be an FDR q value of the GSEA test < 0.05.

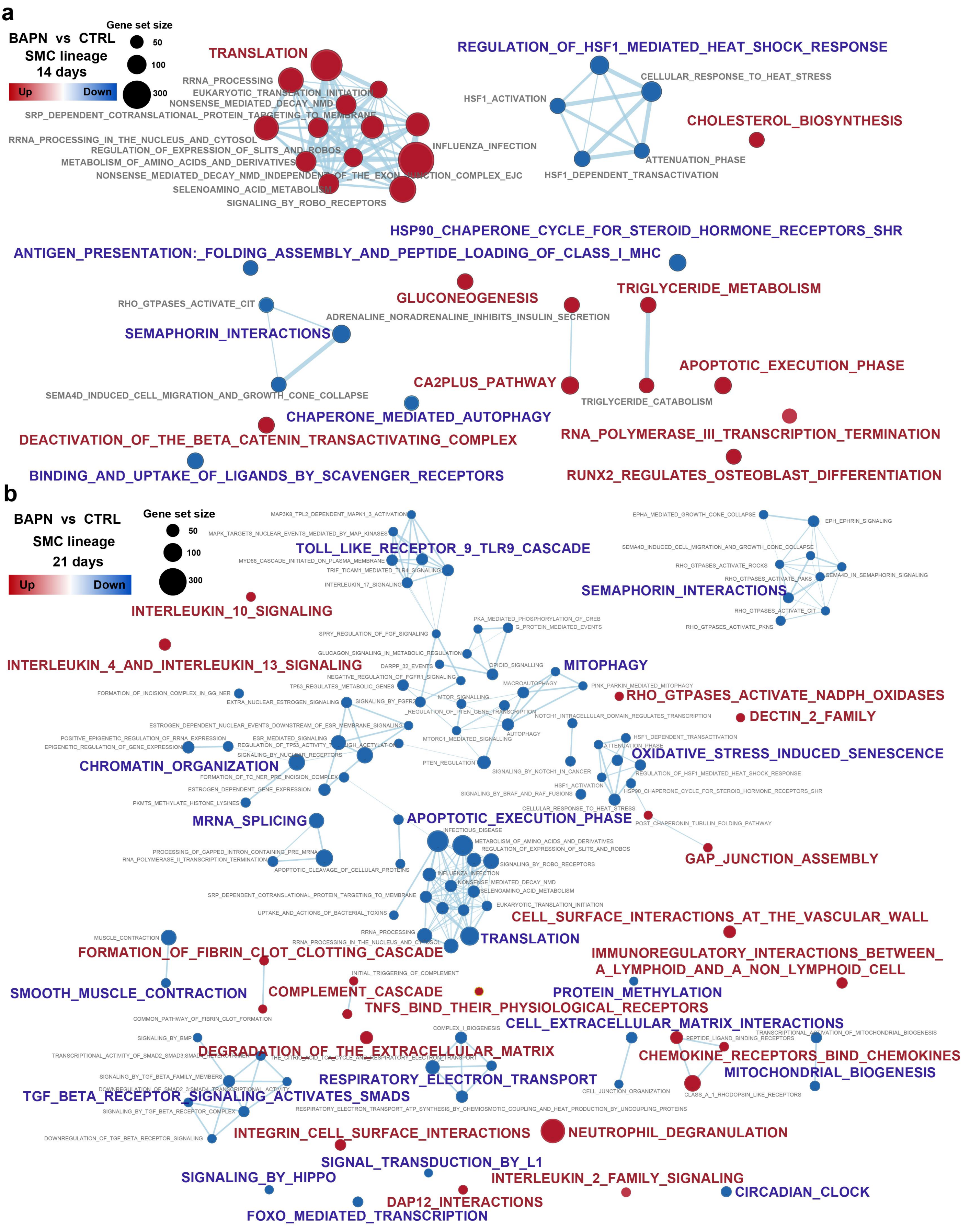

Fig. S4. Network view of the differentially regulated REACTOME pathways in SMCs after 14 or 21 days of BAPN administration. a Differentially regulated REACTOME pathways in SMCs after 14 days of BAPN administration. **b** Differentially regulated REACTOME pathways in SMCs after 21 days of BAPN administration. The size of the dot reflects the size of the gene set. The dots in red denote up-regulated pathways, and dots in blue represent down-regulated pathways. The significance threshold of the GSEA test

was set to an FDR q value of 0.05. The pathways shown are representative ones that were upregulated during the development of TAAD.

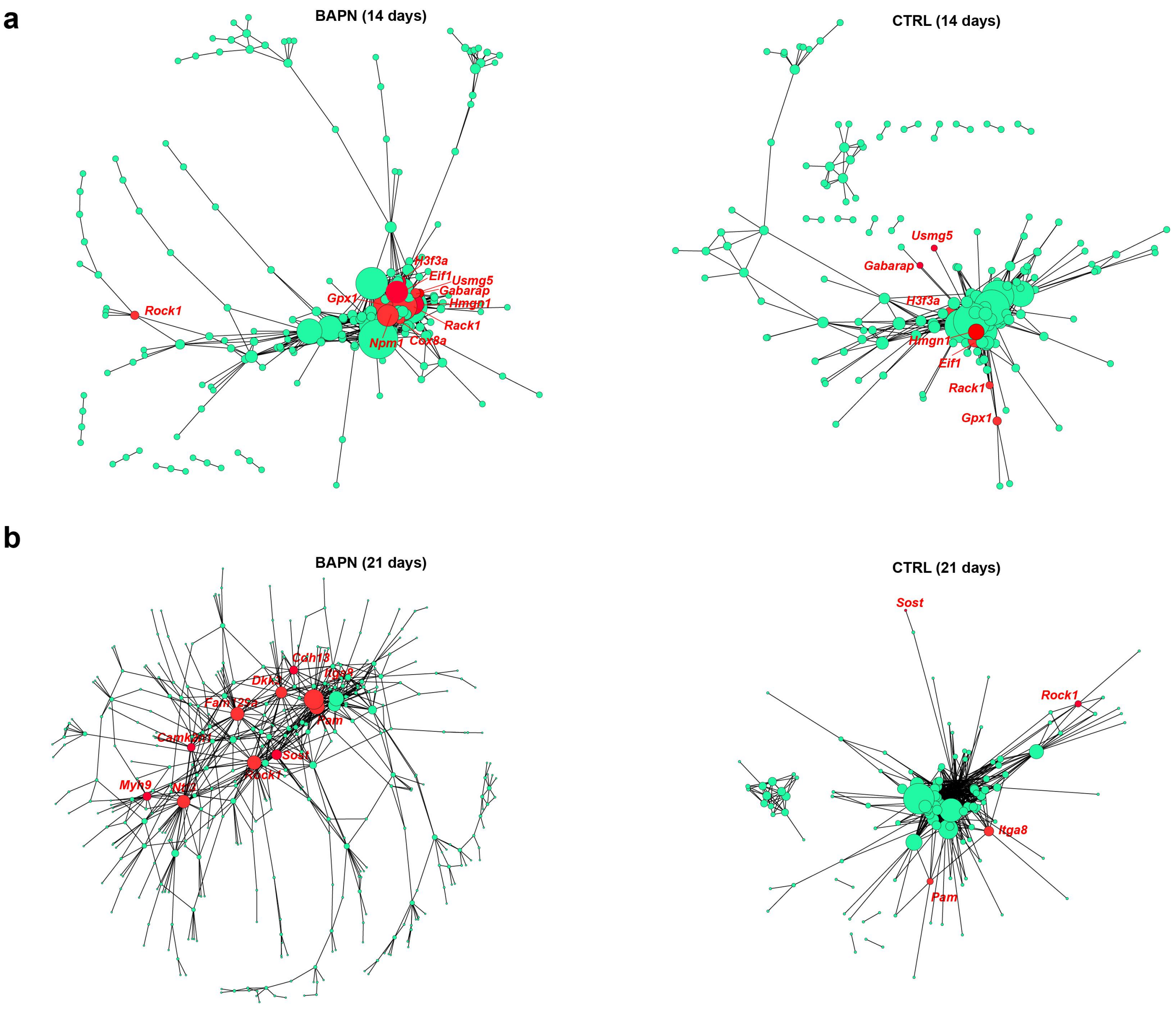

Fig. S5. Comparative analysis of the gene regulatory networks of SMCs between BAPN and CTRL reveals dysregulated genes after 14 or 21 days of BAPN administration. a The gene regulatory networks of SMCs in BAPN (left panel) and CTRL (right panel) after 14 days of administration. **b** The gene regulatory networks of SMCs in BAPN (left panel) and CTRL (right panel) after 14 days of administration. The node size reflects degree centrality. The top genes ranked by delta degree are colored in red.

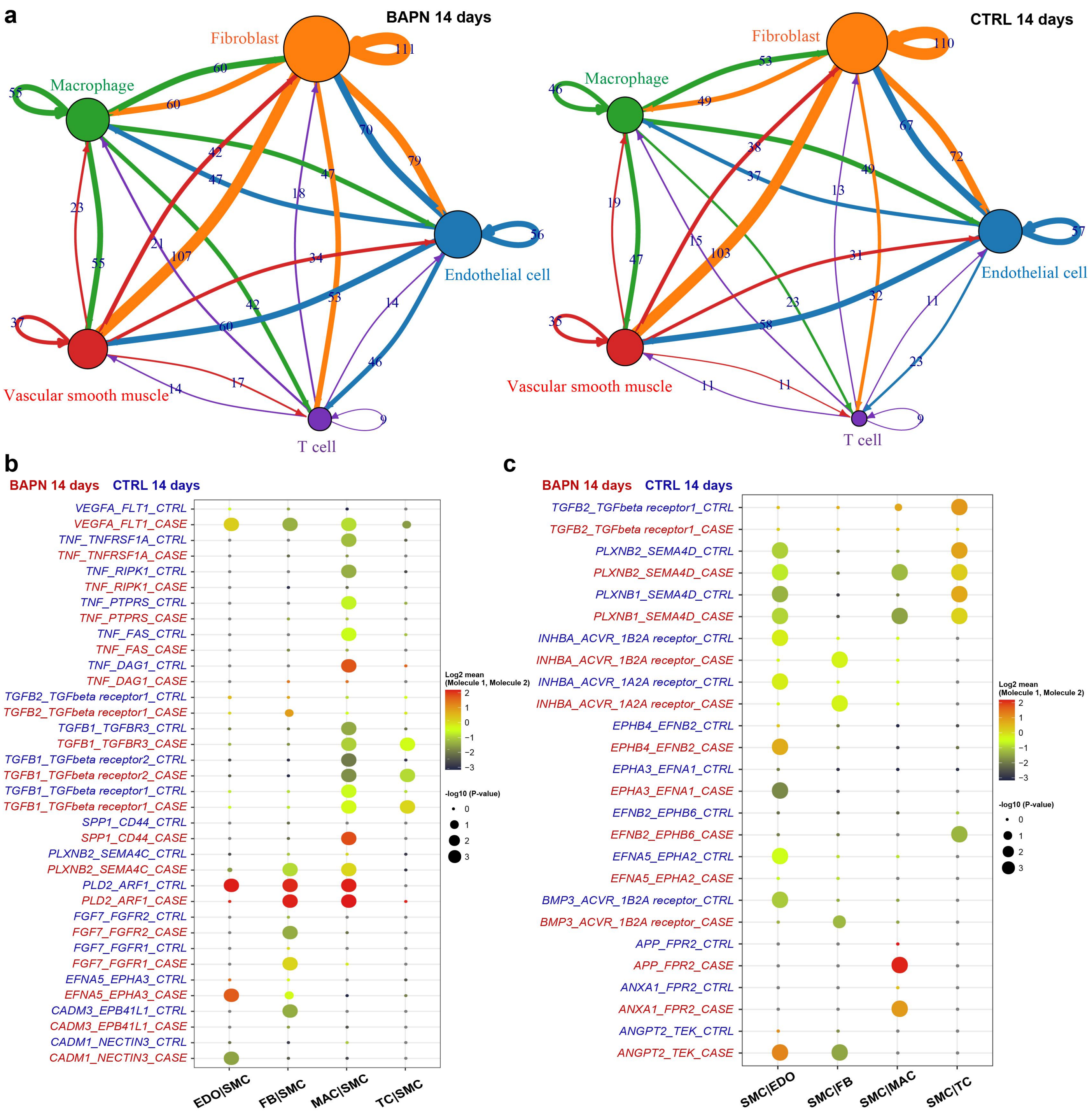

Fig. S6. Cell-cell communication change in the mouse aortic tissue after 14 days of BAPN administration. a Cell-cell communication networks in aortic tissues of BAPN (left panel) and CTRL (right panel). The node size reflects the total number of communications for each lineage. The line color indicates that the ligands are broadcast by the cell lineage in the same color. The line thickness is proportional to the number of broadcast ligands. **b** The ligand-receptor pairs with significant changes in specificity between any one of the non-SMC lineages and SMCs in BAPN versus CTRL at the early stage of TAAD. SMCs express receptors and receive ligand signals from other lineages. **c** The representative ligand-receptor pairs with significant changes in specificity between SMCs and the other lineages in BAPN versus CTRL at the early stage of TAAD. SMCs express ligands and broadcast ligand signals for other lineages. In **b** and **c**, the dot size reflects the *P* value of the permutation tests for lineage-specificity, and the dot color denotes the mean of the average ligand-receptor expression in the interacting lineages. EDO: endothelial cell; FB: fibroblast; MAC: macrophage; SMC: smooth muscle cell; TC: T cell; B cells are not considered due to too few cells.

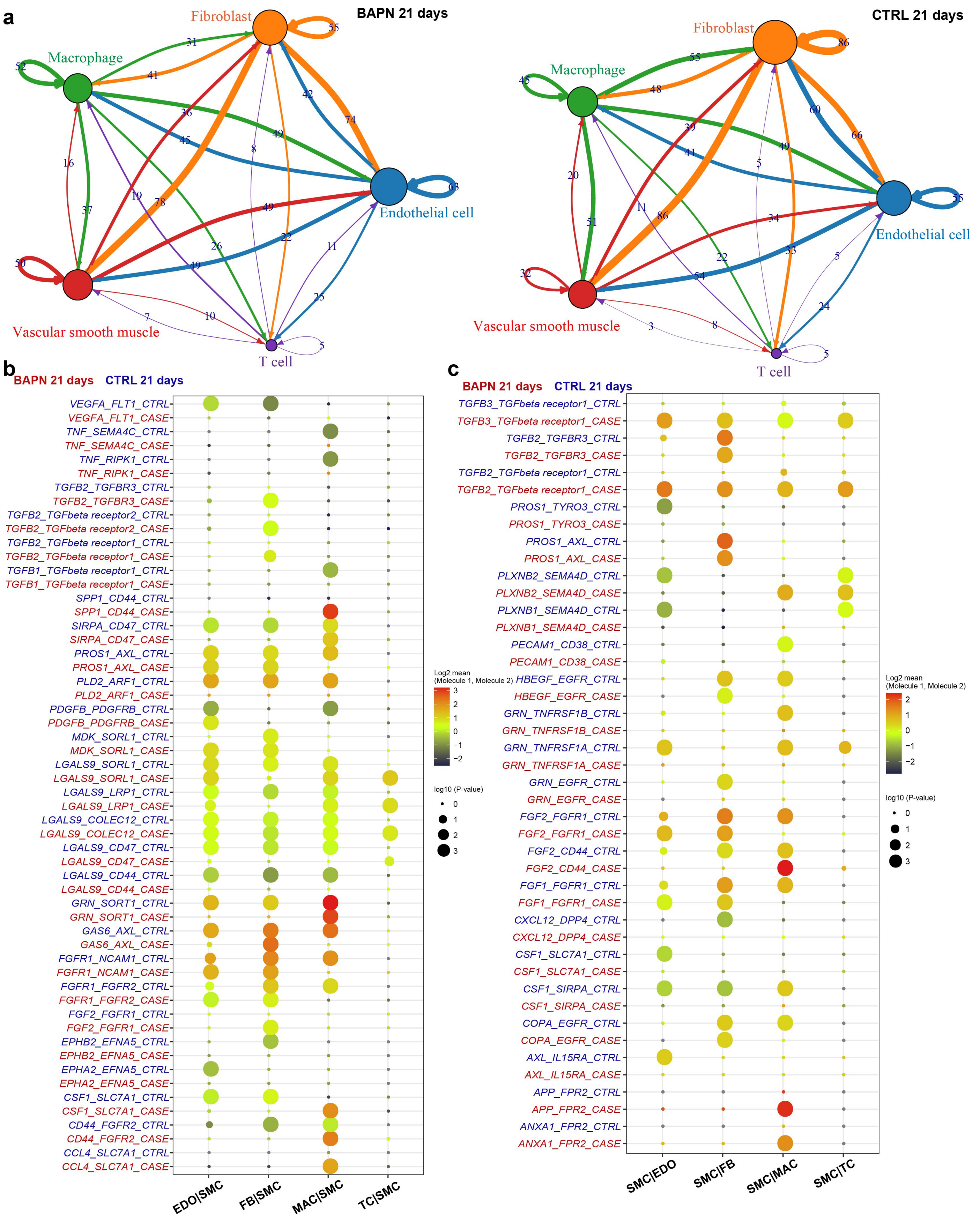

Fig. S7. Cell-cell communication change in the mouse aortic tissue after 21 days of BAPN administration. a Cell-cell communication networks in aortic tissues of BAPN (left panel) and CTRL (right panel). The node size reflects the total number of communications for each lineage. The line color indicates that the ligands are broadcast by the cell lineage in the same color. The line thickness is proportional to the number of broadcast ligands. **b** The ligand-receptor pairs with significant changes in specificity between any one of the non-SMC lineages and SMCs in BAPN versus CTRL at the early stage of TAAD. SMCs express

receptors and receive ligand signals from other lineages. **c** The representative ligand-receptor pairs with significant changes in specificity between SMCs and the other lineages in BAPN versus CTRL at the early stage of TAAD. SMCs express ligands and broadcast ligand signals for other lineages. In **b** and **c**, the dot size reflects the *P* value of the permutation tests for lineage-specificity, and the dot color denotes the mean of the average ligand-receptor expression in the interacting lineages. EDO: endothelial cell; FB: fibroblast; MAC: macrophage; SMC: smooth muscle cell; TC: T cell; B cells are not considered due to too few cells.

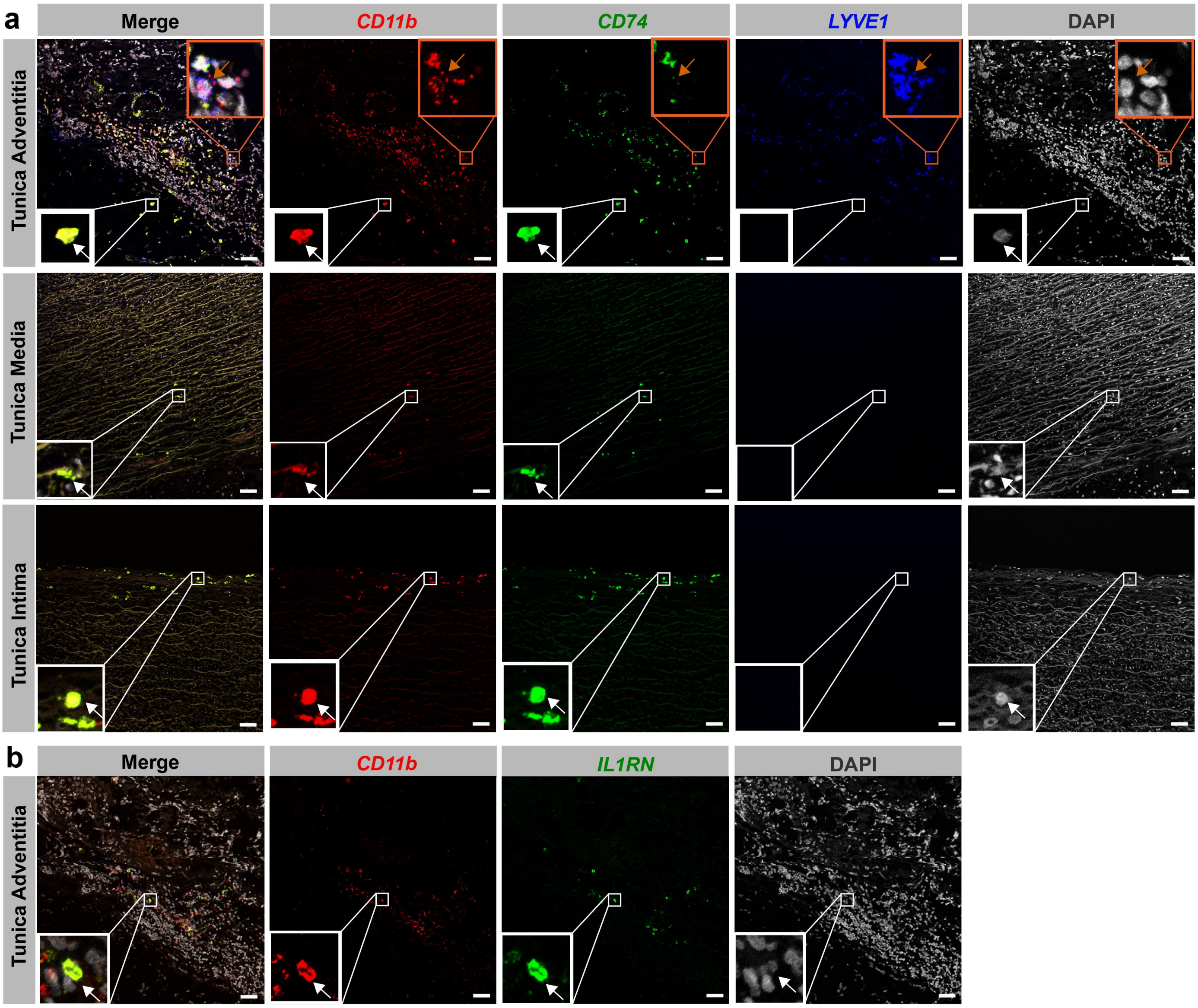

Fig. S8. smFISH showing the spatial distribution of the three macrophage subpopulations in aortic tissue of human TAAD patients. a The *LYVE1* $^{+}$ macrophage subpopulation was localized only in tunica adventitia and the *CD74* ^{high} subpopulation was localized in all the three layers of the aorta. Orange arrows indicate *LYVE1*$^{+}$ *CD74* ^{low} *CD11b* $^{+}$ cells. White arrows indicate *CD74* ^{high} *CD11b* $^{+}$ cells. **b** The *IL1RN* $^{+}$ subpopulation was localized only in tunica adventitia. White arrows indicate *IL1RN* $^{+}$ *CD11b* $^{+}$ cells. Scale bar: 50 µm

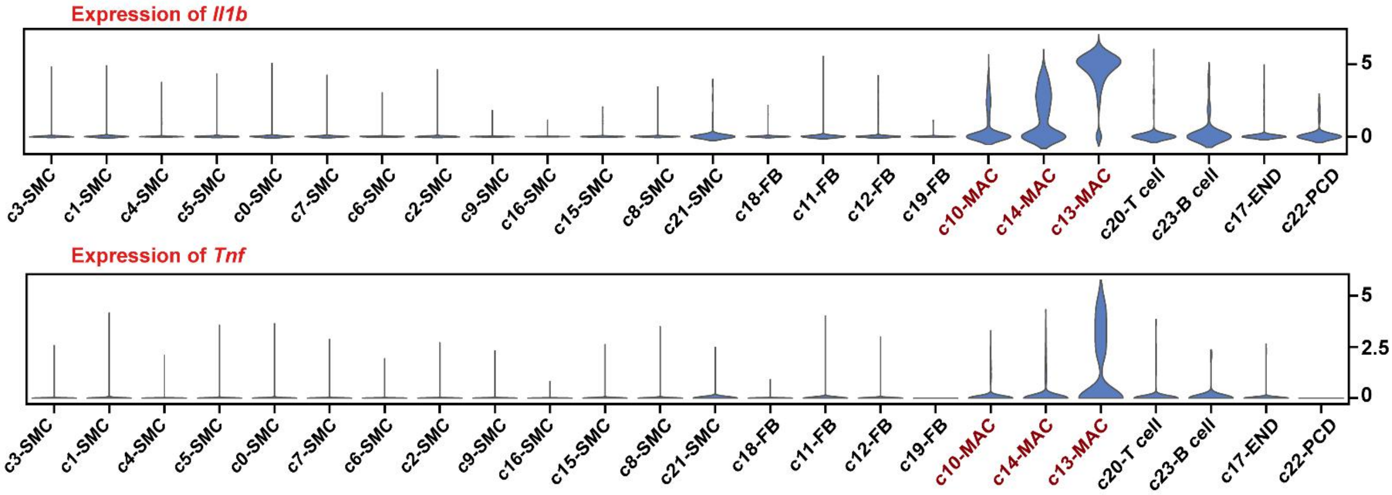

Fig. S9. *Il1b* and *Tnf*, two genes encoding detrimental cytokines for the development of TAAD, are predominately expressed by the proinflammatory macrophage subpopulation c13. EDO: endothelial cell; FB, fibroblast; MAC: macrophage; PCD: pericardial cell; SMC: smooth muscle cell

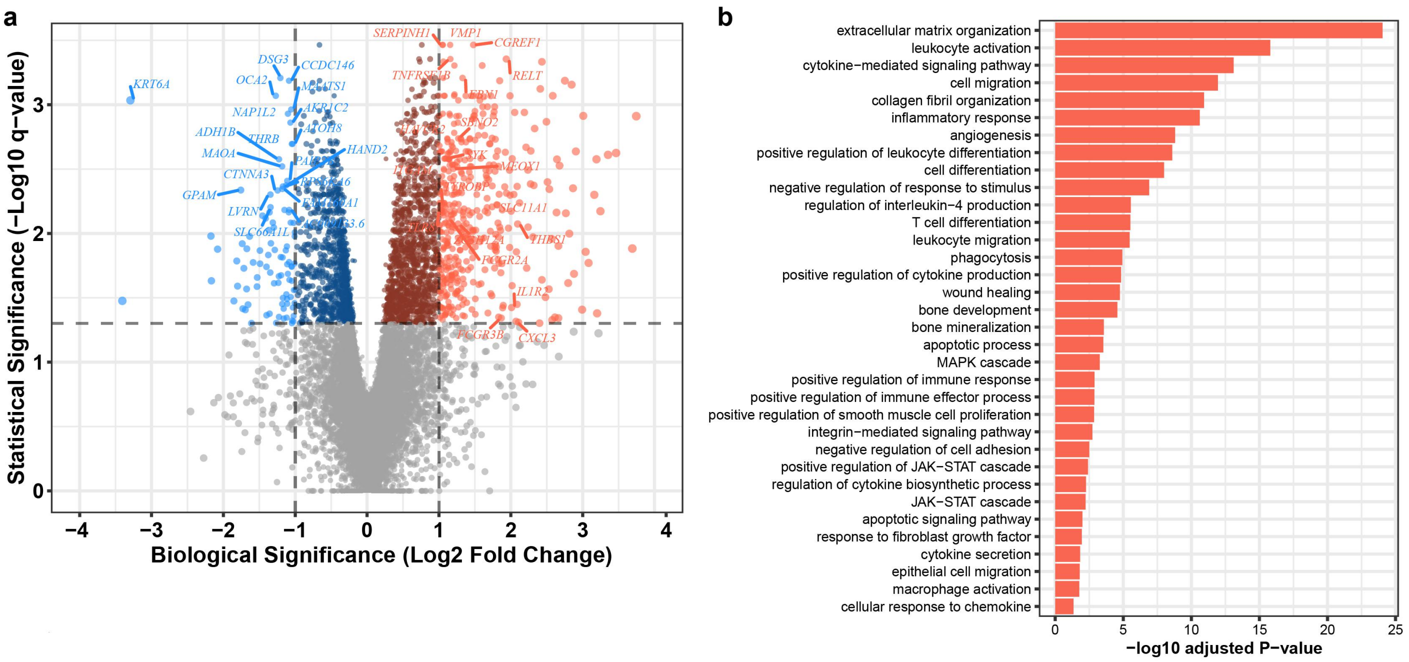

Fig. S10. Differentially expression analysis between aortic tissue from TAAD patients (*n* = 8) and healthy donors (*n* = 6). a Volcano plot showing the differentially expressed genes. The statistically significance threshold was set to be a q value < 0.05 and the biologically significance threshold was set to be an absolute value of log2 fold change > 1. **b** Functional enrichment analysis of the upregulated genes in aortic tissue of TAAD compared with healthy conditions.

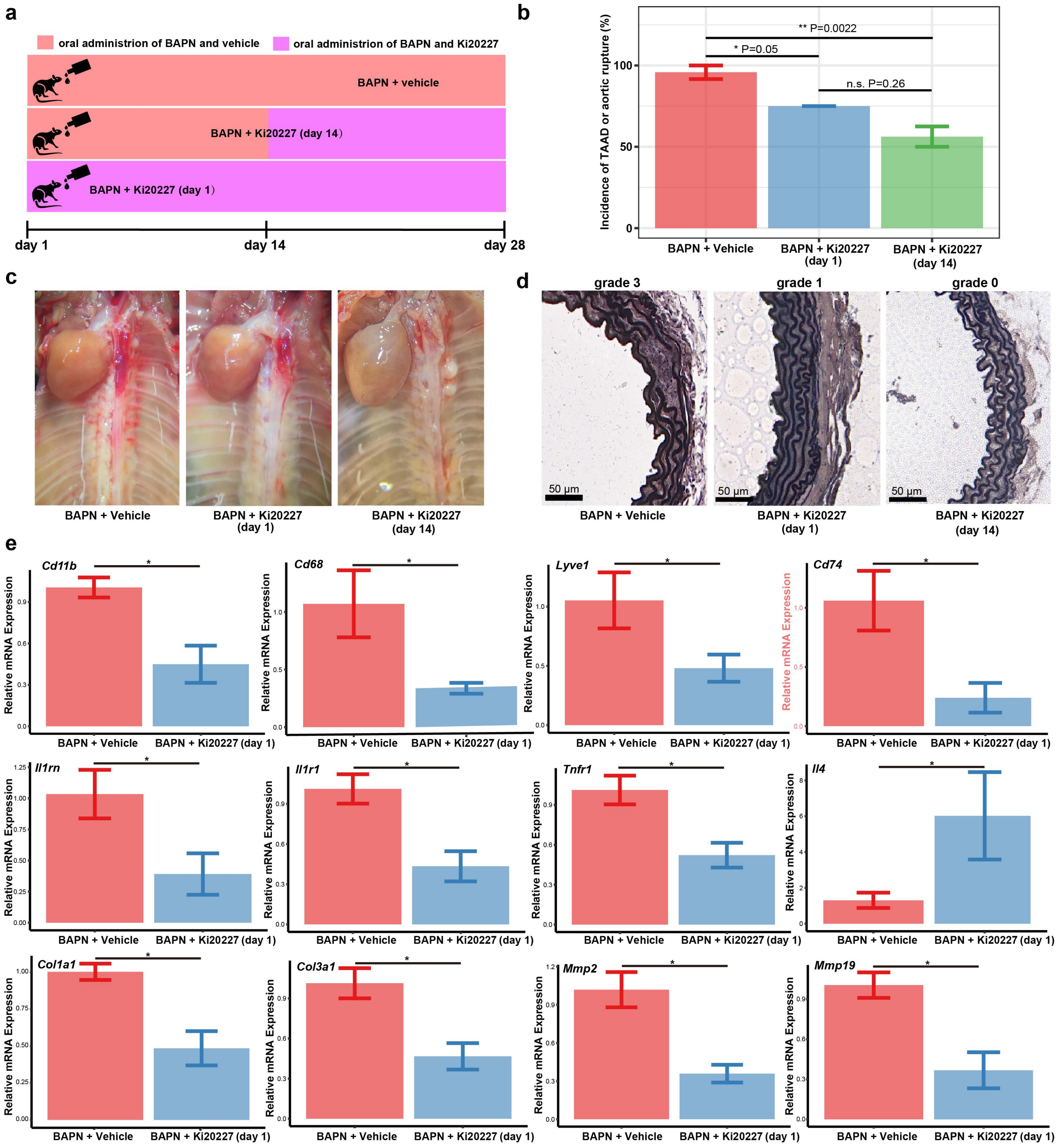

Fig. S11. Depletion of macrophages with an inhibitor Ki20227 could significantly decrease the incidence of TAAD and aortic rupture in mice. a Schematic representation of the experimental procedure. **b** Ki20227 could significantly decrease the incidence of TAAD and aortic rupture. The incidence was examined after 28 days of treatment. Data are represented as individual values with mean ± SEM bars. Three biological replicates were performed for each treatment. Eight mice (n = 8) were considered for each replicate. The statistical significance threshold was set to a Pearson's χ^2 test P value ≤ 0.05. **c** Ki20227 treatment could alleviate the phenotype of TAAD. The thoracic aortas of representative mice with TAAD are shown. **d** Elastin staining showing reduced elastic fiber degradation by Ki20227 treatment. Elastin degradation was graded as follows: grade 0 (intact fibers with normal physiological curvature), grade 1 (stretched fibers with lost physiological curvature), grade 2 (a few fragmented fibers observed), and grade 3 (severely destructed fibers). **e** The expression changes of genes related to the pathogenesis of TAAD in

response to Ki20227 treatment. Relative expression levels were assayed using qPCR. Thoracic aortas from 3-4 mice were used for each treatment. * $P \leq 0.05$, Wilcoxon rank sum test.

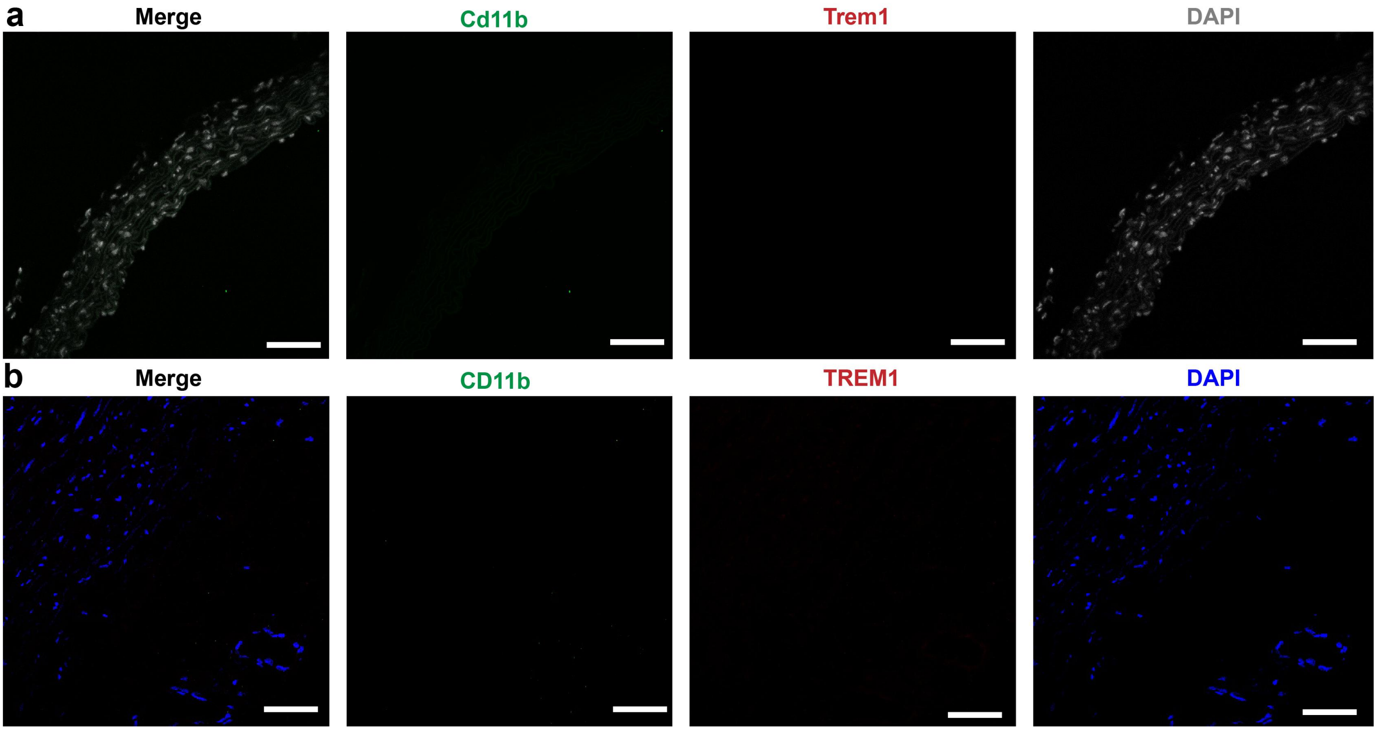

Fig. S12. Negative control for staining IgG in the aortic tissues. a IgG staining in the aortic tissues of BAPN-induced TAAD mice. **b** IgG staining in the aortic tissues of human TAAD patients. Scale bar: 50 μm

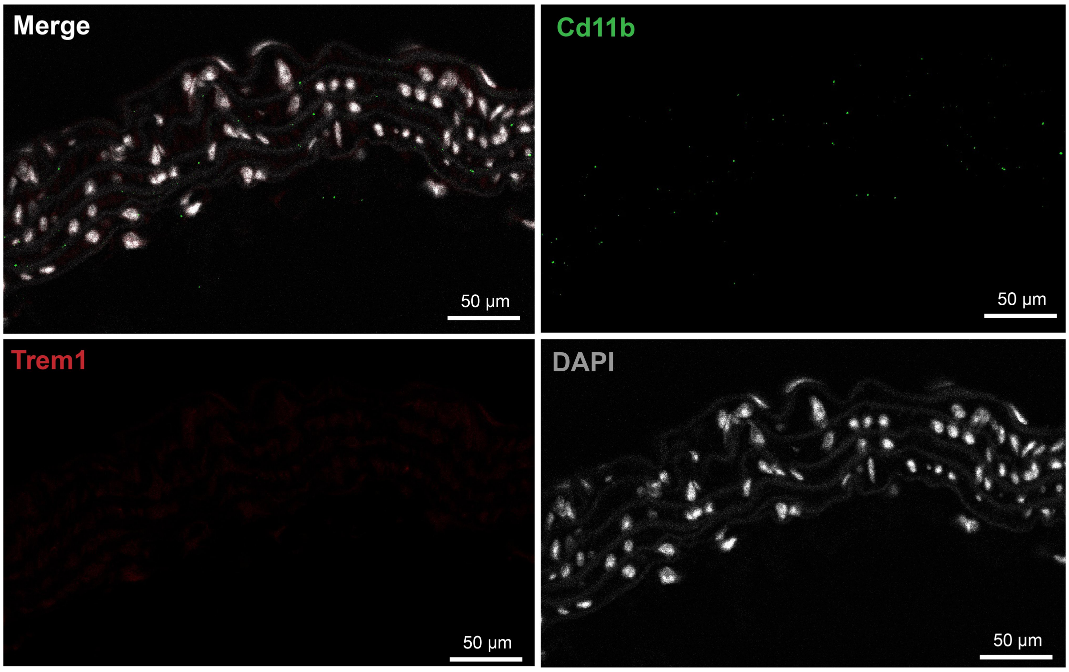

Fig. S13. Immunofluorescence staining identifies few Cd11b$^+$ Trem1$^+$ cells in aortic tissues of the healthy control group mice. Scale bar: 50 μm
